# Proteome and Secretome Profiling of the Melanoma-Induced Transition Toward Immune Incompetent Dendritic Cells Reveals Enhanced IDO1, Cathepsin, and Legumain Activity

**DOI:** 10.1016/j.mcpro.2025.101048

**Published:** 2025-08-09

**Authors:** Anouk M.D. Becker, Bob J. Ignacio, Jelmer J. Dijkstra, Alexander R. Ziegler, Iván Ramos-Tomillero, Floris J. van Dalen, Laura E. Edgington-Mitchell, Michiel Vermeulen, Kimberly M. Bonger, I. Jolanda M. de Vries, Martijn Verdoes

**Affiliations:** 1 Medical BioSciences, Radboud University Medical Center, Nijmegen, The Netherlands; 2 Department of Biochemistry & Pharmacology, Bio21 Molecular Science and Biotechnology Institute, The University of Melbourne, Parkville, Australia; 3 Department of Synthetic Organic Chemistry, Chemical Biology Lab, Radboud University, Nijmegen, The Netherlands; 4 Department of Molecular Biology, Faculty of Science, Oncode Institute, Radboud University Nijmegen, Nijmegen, The Netherlands; 5 Division of Molecular Genetics, The Netherlands Cancer Institute, Amsterdam, The Netherlands; 6 Institute for Chemical Immunology, Radboud University Medical Center, Nijmegen, The Netherlands; 7 Chemical Biology & Immunology, Leiden Institute of Chemistry, Leiden University, Leiden, The Netherlands

## Abstract

Dendritic cells (DCs) are professional antigen-presenting cells endowed with the capacity to initiate strong antitumor immune responses. This function is critical for effective DC-based immunotherapies but is often hampered by tumor-derived immunosuppressive factors, as is observed for CD14^+^CD163^+^ tumor-induced DC3s (ti-DC3s). ti-DC3s are increased in cancer patients where they display an immunosuppressive phenotype, accompanied by weak antigen-specific CD8 T cell–activating capacities. While tumor-derived interleukin-6, macrophage colony-stimulating factor, and prostaglandin E2 have been identified as factors inducing the transition from DC2s to ti-DC3s, a comprehensive unbiased profiling of the resulting changes in secretome and proteome has not been reported. Here, we characterized by tandem LC-MS/MS the proteomic changes in conventional DCs during their transition into CD14^+^ ti-DC3s *in vitro*, using conditioned medium from the melanoma cell line BLM. This revealed 157 differentially expressed proteins, including upregulated indoleamine-2,3-dioxygenase 1 and legumain, which we confirmed to be functionally active. Next, we profiled the newly synthesized secretome in human DCs with THRONCAT metabolic labeling. We detected 17 differentially secreted proteins between DC2s and ti-DC3s, which included six cathepsins and tumor-associated transforming growth factor-β–induced protein. Cathepsin activity was validated in peripheral blood and tumor tissue of melanoma patients. We detected the highest cathepsin activity in ti-DC3s, surpassing DC2s and tumor-associated macrophages. Together, our findings represent the first characterization of the proteome and secretome of human melanoma-induced DC3s. This revealed several protein-driven protumor mechanisms active in ti-DC3s that potentially contribute to creating an immune environment favorable for tumor progression.

## Highlights

• Secretome and proteome dataset of tumor-induced changes in human dendritic cells.
• First application of a novel metabolic labeling method in dendritic cells.
• Identification of protein driven protumor mechanisms employed by dendritic cells.
• Validation of our findings in peripheral blood and tumor tissue of cancer patients.

## Graphical Abstract

**Figure:**
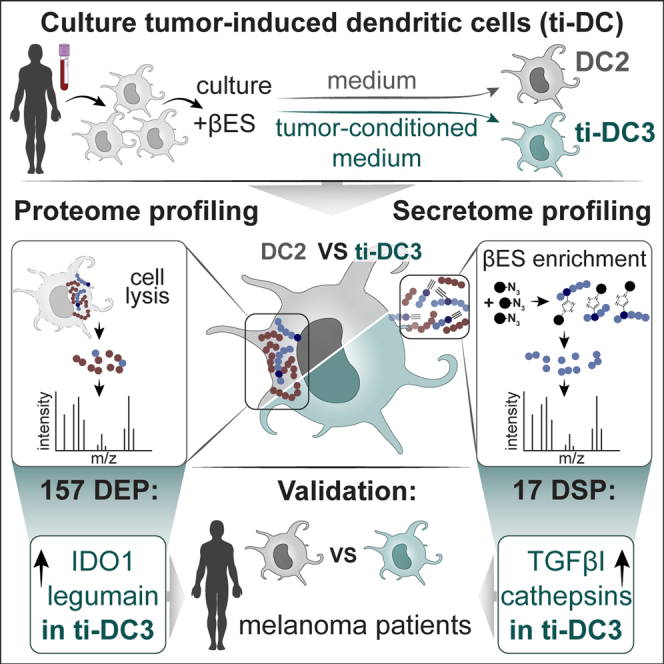

## In Brief

Tumors secrete factors that impair dendritic cell (DC) function, critical for antitumor immunity. We employed mass spectrometry–based proteomics to profile the tumor-induced changes in DCs. Among the 157 differentially expressed proteins, IDO1 and legumain were upregulated. Furthermore, we pioneered the application of the metabolic labeling technique THRONCAT in DCs. This enabled characterizing the newly synthesized secretome, which uncovered upregulated cathepsins. Validation in cancer patients underscored the relevance and applicability of THRONCAT to obtain a deeper understanding of important cellular processes.

Dendritic cells (DCs) are professional antigen-presenting cells pivotal in orchestrating the immune response between innate and adaptive immunity. DCs are specialized in antigen uptake and subsequent initiation of an immune response (1). Based on their specialized functions, ontogeny, phenotype, and transcriptome, human blood DCs are divided in plasmacytoid DCs (pDC), type 1 conventional DCs (cDC1), and type 2 conventional DCs (cDC2) (2). cDC2s are a heterogenous subset, subdivided in DC2 (CD1c^+^CD5^+/−^CD14^−^CD163^−^) and DC3, phenotypically defined as CD1c^+^CD14^+^CD163^+^ (2).

DC-based immunotherapies such as patient-derived DC vaccinations take advantage of the ability of DCs to initiate potent antitumor immune responses leading to eradication of tumor cells. However, a major hurdle in developing effective immunotherapies is the tumor microenvironment, which contains a myriad of immunosuppressive cellular and soluble factors. For stage III and IV melanoma patients receiving CD1c^+^ DC-based vaccinations, the presence of CD1c^+^CD14^+^ DCs (DC3) in vaccine preparations suppresses CD4^+^ T cells (3, 4). Moreover, melanoma patients and non–small cell lung cancer patients have increased levels of DC3s in peripheral blood (3, 4). These DC3s display an immunosuppressive phenotype characterized by high levels of programmed death ligand 1 (PD-L1), MER proto-oncogene tyrosine kinase, interleukin (IL)-10, and indoleamine-2,3-dioxygenase 1 (IDO1). Functionally, CD14^+^ DC3s are inferior tumor-antigen–specific CD8^+^ T cell activators compared to CD14^−^ DC2s. Further investigation into CD14^+^ DC3s revealed that they can originate from CD14^−^ DC2s, driven by tumor-secreted IL-6 and macrophage colony-stimulating factor (M-CSF), hence called tumor-induced CD14^+^ DC3s (4). Although apparent that CD14^+^ DC3s are expanded in cancer patients, the underlying mechanisms contributing to their protumor effects and immune incompetence remain to be explored.

Many biological processes (BPs), and thereby functional abilities of cells, are regulated by the abundance of expressed proteins (5, 6, 7). Protein expression is determined by several factors in addition to mRNA levels, including translation rates depending on mRNA sequence and modulation, protein half-life, synthesis delays, and protein transport. Accordingly, poor correlations are reported between transcriptome and proteome (5, 6). Part of the proteome (13–20%) is released into the extracellular space after synthesis and constitutes the secretome of the cell (8). Proteins of the secretome are often important effector molecules, such as cytokines, chemokines, growth factors, and extracellular matrix (ECM) proteins, participating in vital functions (8). To be able to rapidly respond to environmental changes, immune cells such as NK cells, memory T cells, and myeloid cells, constitutively express mRNA encoding for such effector molecules (9, 10, 11). Whether these are produced and secreted is tightly regulated by posttranscriptional mechanisms, including RNA-binding proteins capable of repressing mRNA translation, further underlining the necessity of proteomics to supplement transcriptomics (12, 13). Therefore, proteomic profiling combined with an analysis of the secretome will provide fundamental knowledge to understand the mechanisms shaping the functional abilities of DCs.

**Fig. 1:**
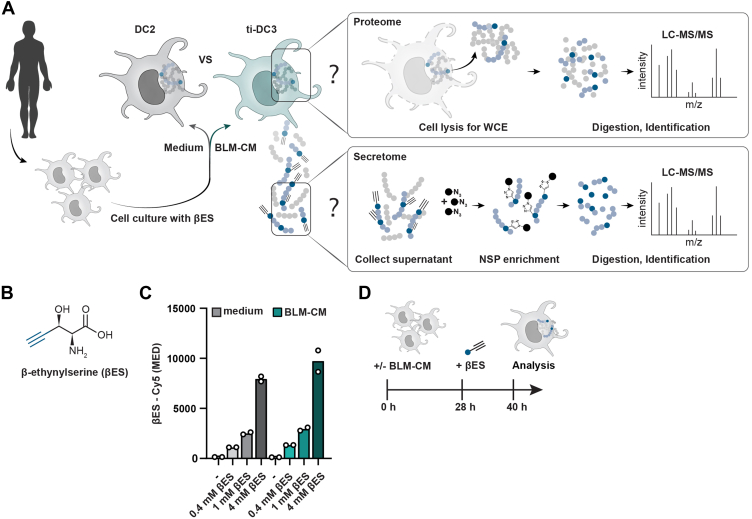
**Proteome and secretome profiling in primary human DC cultures.***A,* this study aims to investigate proteomic and secretomic changes during human primary DC2 transition into tumor-induced DC3s (ti-DC3). Differences in the proteome will be assessed by single shot proteomics with label-free quantification using LC-MS/MS. To compare the secretome, newly synthesized proteins (NSPs) will be selectively enriched from supernatant using conjugation of the alkyne in βES to azide agarose beads, prior to analysis by LC-MS/MS. *B,* structure of the bioorthogonal threonine analog β-ethynylserine (βES) used in all metabolic labeling experiments, with the alkyne group used for azide-alkyne cycloaddition reaction depicted in *blue*. *C,* median (MED) fluorescent signal of βES-Cy5 after 1 h of metabolic labeling with increasing concentrations of βES in X-VIVO medium with and without 50% BLM-conditioned medium (CM). Each *circle* represents a technical replicate, *bars* represent mean values (N = 1). *D,* schematics of the final timeline in which cDC2s are cultured with or without BLM-CM for 28 h prior to labeling for 12 h with βES, leading to a total culture period of 40 h. See also Supplemental Figure S1. cDC2, type 2 conventional dendritic cell.

In addition to quantitative whole-cell proteomics, approaches have been developed to specifically identify newly synthesized proteins (NSPs). These approaches enrich NSPs to decrease sample complexity and facilitate the identification of low-abundance proteins (7). Additionally, the NSP isolation can specifically enrich cell-derived proteins, the secretome, from complex mixtures such as culture media proteins. A well-established method is bioorthogonal noncanonical amino acid tagging (BONCAT) (7). In BONCAT, cells are cultured with a methionine (Met) analog that is incorporated during protein synthesis into the sites normally occupied by this amino acid. The analog contains a bioorthogonal chemical reactive group (typically an azide or alkyne functionality), enabling the conjugation to reporter molecules for visualization or enrichment of NSPs (7, 14). However, employing BONCAT to investigate NSPs in primary DCs is hindered by technical demands. First, the Met surrogates require Met-free culture conditions for efficient incorporation. Met starvation impedes protein translation, posttranslational modifications, and cell cycle progression, thereby strongly affecting the native cell dynamics (15, 16). Moreover, DCs require specialized medium to support optimal culture and are highly sensitive to changes in culture medium (17). Any starvation-induced cell toxicity would affect cell numbers and DC maturation through released damaged-associated molecular patterns. Collectively, this challenges the applicability of BONCAT to analyze the secretome of human DCs. The recently developed metabolic labeling approach threonine-derived noncanonical amino acid tagging (THRONCAT) (18) could overcome these challenges. THRONCAT is based on the bioorthogonal threonine (Thr) analog β-ethynylserine (βES, Fig. 1, *A* and *B*). βES competes with Thr in complete culture medium, providing the opportunity to metabolically label DCs without the need for starvation and medium adjustments. Rapid and efficient labeling of mammalian cell lines without noticeable toxicity further supports the utility of THRONCAT for primary cells. Altogether, the improvements in THRONCAT facilitate metabolic labeling in DCs, allowing the first characterization of the nascent secretome from primary human cells.

In this work, we analyzed changes in the proteome and secretome of human cDC2s during their tumor-induced conversion to CD14^+^ DC3s by quantitative whole-cell proteomics and THRONCAT. This presents for the first time a comprehensive, unbiased profiling, fundamental to gain a deeper understanding of tumor-induced DC3. The whole-cell proteomics detected 157 differentially expressed proteins, including upregulated IDO1 and legumain (LGMN) in tumor-induced DC3s. THRONCAT was successfully applied to examine the secretome of DCs, which opens new possibilities to examine protein synthesis and secretion during important BPs in primary immune cells. Finally, we supplemented our proteome and secretome profiling with measurements of IDO1, LGMN, and cathepsin activity in DC2s and DC3s, including tumor tissue samples from melanoma patients. Altogether, our work provides new insights in processes at the protein level that affect the protumor and immunostimulatory capability of melanoma-induced CD14^+^ DC3s.

## Experimental Procedures

### Experimental Design and Statistical Rationale

To profile the proteome and secretome changes of DC2 transitioning to tumor-induced DC3 (ti-DC3), an *in vitro* model utilizing DC2s isolated from healthy donors (HD) was employed. Apheresis material obtained from five healthy adult donors was pooled to account for donor variability and achieve high enough cell numbers to yield sufficient protein amounts. This material was divided over the experimental condition (BLM-conditioned medium [CM]) and the control condition (medium), with each condition consisting of three technical replicates. All samples were processed under identical conditions except for the factor under investigation. Statistical analysis of LC-MS/MS data was performed using the differentially expressed protein (DEP) package with false discovery rate (FDR) correction using “fdrtools.” Single-shot proteomics consisted of three technical replicates; secretome analysis consisted of the forward and the reverse run. To ensure data reliability, proteins identified by only a single unique peptide were excluded. Details of the analysis are outlined in the respective method section below.

The proteome and secretome analysis were followed by several experiments aimed at validating detected targets, using HDs or cancer patient material. Each corresponding figure legend contains the exact sample size as a number and a clear indication of the replicate type (biological *versus* technical). The statistical method was decided based on a normal distribution and the number and direction of comparisons required for the specific analysis (multiple group comparisons were done with one-way or two-way ANOVA, followed by correction for multiple testing). Each figure legend therefore indicates the specific statistical analysis employed. Results are analyzed in GraphPad Prism software (V8) for Windows, GraphPad Software, www.graphpad.com, and presented as mean ± SD in scatter plots (with bars), unless otherwise indicated. Statistical significance was annotated as: ∗*p* < 0.05, ∗∗*p* < 0.01, and ∗∗∗*p* < 0.001.

### Human Blood Immune Cells

Human peripheral blood mononuclear cells (PBMCs) were obtained from buffy coats (Sanquin) or apheresis material (Tumor Immunology department, Radboudumc) from healthy volunteers that provided informed consent. PBMCs were obtained from seven stage IV melanoma patients who had participated in clinical studies (19, 20).

All studies from which human material is included are conducted according to the principles of the Declaration of Helsinki and adhere to the Dutch Medical Research Involving Human Subjects Act (WMO) and Good Clinical Practice guidelines. Patient information is depicted in Supplemental Table S8. Approval was granted by the Medical Ethical Committee Arnhem-Nijmegen (CMO). The patient from whom melanoma brain metastasis tissue was obtained signed informed consent prior to surgery and is part of a study for which ethical approval was obtained under CMO Radboudumc; 2022-13427. PBMCs were isolated using Lymphoprep (Axis-Shield PoC AS). CD14^+^ monocytes were isolated directly from PBMCs using CD14 MACS microbeads combined with FcR Blocking Reagent (Miltenyi). PBMCs used for isolation of CD1c^+^CD14^−^ cells were depleted for CD3, CD56, CD19, and CD14 using MACS microbeads and LD columns prior to positive selection of CD1c using the CD1c (BDCA1) DC isolation kit (Miltenyi) following manufacturer's protocol. Purity was assessed by staining with CD1c-BV421, CD14-APC-H7, CD20-FITC, CD3-PE, and acquisition on BD FACSVerse or BD FACSLyric (Supplemental Fig. S2*B*).

### Cell Line Culture

Human melanoma BLM cells were obtained from AIMM Therapeutics and HeLa cells from American Type Culture Collection (www.atcc.org), both tested *mycoplasma* free. Cell lines were maintained by culture in Dulbecco's modified Eagle medium (GIBCO) supplemented with 10% heat inactivated fetal bovine serum and 1% Antibiotic-Antimycotic (Thermo Fisher Scientific) in humidified incubators at 37 °C and 5% CO_2_. To obtain tumor-CM from BLM cells, supernatant was collected after a 4-day culture period, centrifuged at 1500 rpm for 5 min to pellet remaining cells and cell debris, aliquoted, and stored at −20 °C until further use.

### Culture of cDC2s and Monocytes

For all cell culture experiments followed by flow cytometry analysis, between 50,000 and 100,000 cDC2s were cultured in 200 μl X-VIVO 15 (Lonza) with 2% human serum (HS) (Sigma-Aldrich) in round-bottom 96-well plates for indicated time periods. Conversion of cDC2s to CD14^+^ cDC2s was induced by culturing cDC2s in 50% X-VIVO 2% HS and 50% BLM-CM (total volume 200 μl) for the indicated time periods. For mass spectrometry experiments, monocytes were isolated from a buffy coat and cDC2s from apheresis material from five HDs. Cells were cultured in T75 flasks with 1·10^6^ cells/ml. For cDC2s, 23.6·10^6^ cells were divided over two T75 flasks for the medium condition and 19.3·10^6^ cDC2s over two T75 flasks for the condition with 50% BLM-CM. All cells were cultured in humidified incubators at 37 °C and 5% CO_2._

### Metabolic Labeling of cDC2s and HeLa Cells for Flow Cytometry

Metabolic labeling reporter βES was kindly provided by Bob Ignacio (Bonger lab, Radboud University Nijmegen) and synthesized as previously described (18). HeLa cells were seeded at a density of 45,000 cells per well in a 48-well plate and cultured for 1 day prior to 1 h βES incubation in various concentrations. cDC2s were incubated with βES for various time points and concentrations, as indicated in corresponding figures. After incubation, cells were harvested and analyzed by flow cytometry.

### Flow Cytometry

Antibodies used for flow cytometry with corresponding dilutions are all listed in Supplemental Table S6 and used panels in Supplemental Table S7. Briefly, flow cytometry stainings were performed in V-bottom 96-well plates (Greiner BioOne) at 4 °C protected from light. Cells were washed with PBS and incubated with eBioscience Fixable Viability Dye e506 (Thermo Fisher Scientific, 1:2000) for 30 min in PBS, followed by two more washes. Cells were incubated for 15 min with FcR blocking reagent (Miltenyi, 1:10) prior to staining with directly labeled primary antibodies for 30 min. When cell viability was analyzed using propidium iodide solution (Miltenyi, 1:100) instead of FVD e506, 1 μl was added to 100 μl cell suspension immediately before acquisition on the flow cytometer. For the IDO1 panel, after FcR blocking, cells were fixed and permeabilized with BD Cytofix/Cytoperm (BD Biosciences) according to the manufacturer's instructions, prior to the intracellular staining with directly labeled primary antibodies for 30 min at 4 °C.

To analyze THRONCAT labeling by flow cytometry, an intracellular staining was performed to conjugate Cy5 to βES *via* copper-catalyzed azide-alkyne cycloaddition (CuAAC) (cells were not stained with additional antibodies). In brief, cells were fixed and permeabilized with BD Cytofix/Cytoperm (BD Biosciences) and incubated for 15 min with blocking buffer (BD perm/wash 1x buffer supplemented with human FcR blocking reagent (Miltenyi, 1:50) and 2% HS. Next, the CuAAC reaction was performed in 100 μl CuAAC buffer (4 mM CuSO_4_· 5 H_2_O, 200 μM Tris-Hydroxypropyltriazolylmethylamine, 0.5 μM sulfo-Cy5-azide (Jena Bioscience) and 40 mM sodium ascorbate with 0.1% saponin in PBS) for 30 min at 37 °C. Cells were subsequently washed three times with 200 μl BD perm/wash buffer and resuspended in 100 μl PBS with 3% bovine serum albumin (Sigma-Aldrich) for acquisition on the flow cytometer. All conditions, including negative controls, were subjected to identical CuAAC labeling conditions.

Anti-mouse Ig, κ/Negative Control Compensation Particles Set (BD) was used for single stain controls. Flow cytometry acquisition was performed on a BD FACSVerse or BD FACSLyric and FlowJo v.10 Software (www.flowjo.com, BD Life Sciences) was used to analyze the data.

### THRONCAT Labeling in Monocytes and cDC2s for Proteomics

Cells were cultured in 1·10^6^ cells/ml X-VIVO (2% HS) or 50% X-VIVO (2% HS) and 50% BLM-CM in T75 flasks for 28 h prior to the addition of βES. βES labeling was performed for 12 h at 37 °C in X-VIVO or 50% X-VIVO and 50% BLM-CM. After incubation, supernatant from cDC2s was collected and centrifuged at 1500 rpm for 5 min to remove cell debris. Cleared supernatant was transferred to 50 ml tubes and stored at −80 °C until further use. For whole-cell extracts, cells were harvested, counted, and indicated cell numbers transferred to Eppendorf tubes. After washing twice with PBS, PBS was aspirated and the cell pellets were snap frozen with liquid nitrogen and stored at −80 °C until further use.

### Proteomics Sample Preparation

To prepare whole-cell extracts, cell pellets were incubated with SDS lysis buffer (4% SDS, 1 mM DTT, 100 mM Tris–HCl pH 7.5 in ultrapure water) for 5 min at 95 °C. Samples were sonicated until homogeneous using alternating cycles of 30 s on/30 s off on high intensity and spun down at 16,000 *g* for 5 min, after which the supernatant was transferred to a new tube. Protein concentrations were determined using a Pierce bicinchoninic acid protein assay (Thermo Fisher Scientific). Afterward, final DTT concentration was corrected to 10 mM. Per sample, 50 μg of total protein was taken for acetone precipitation by adding 10 volumes of ice-cold acetone and incubating for 10′ at −20 °C, followed by spinning for 10′ at 4 °C at max speed. The pellet was washed with 100 μl ice-cold acetone and spinning was repeated. The resulting pellet was resolubilized in 100 μl of 8 M urea (pH 8.0 at room temperature [RT]) and the DTT concentration was adjusted to 10 mM. After 10′ incubation at RT, iodoacetamide was added to a final concentration of 50 mM, followed by 10′ incubation at RT in the dark. Per sample, 0.25 μg Lys-C protease was added and samples were incubated for 2 h at RT, after which 3 volumes of 50 mM ammonium bicarbonate and 0.1 μg trypsin were added, followed by overnight digestion. Next, samples were acidified with 10 to 20 μl TFA and concentrated on StageTips (21).

### Proteomics LC-MS/MS Measurements and Data Analysis

Digested peptide samples were eluted from StageTips with elution buffer (80% acetonitrile [ACN], 0.1% formic acid (FA) in ultrapure water), reduced to 10% of the original volume by vacuum concentration and diluted in 0.1% FA to ∼12 μl. The sample (5 μl) was injected and peptides were separated on an Easy-nLC 1000 liquid chromatography system (Thermo Fisher Scientific) fitted with a new 30 cm objective emitter of fused silica with an inner diameter of 75 μm packed with C18 beads (ReproSil-Pur, 1.9 μm, 120 A) from Dr Maish at a flow rate of 250 nl/min using a 94 min ACN gradient (9–32%), followed by washes with 50% and 95% ACN for a total of 120 min data collection.

Data-dependent measurements of the peptides were performed on a Q-Exactive HF-X mass spectrometer (Thermo Fisher Scientific). Mass spectrometry (MS)1 mass resolution was set to 120,000, the MS1 scan range was 350 to 1300 *m/z*, and tandem mass spectrometry (MS/MS) scan resolution was 15,000. Collision-induced dissociation energy was set at (N)CE 28. Automatic gain control was set at 3.00 × 10^6^ and 1.00 × 10^5^ for MS1 and MS/MS, respectively. The automatic gain control intensity threshold for MS/MS was set at 5.00 × 10^4^. Precursors with charge states of 2 to 5 were selected for fragmentation. For every full scan, the top 20 peptides were selected for fragmentation and dynamic exclusion was set to 30 s with a mass error of 5 ppm. Protein identification and quantification was done in MaxQuant v1.6.0.1 (www.maxquant.org) with default settings, with match between-runs, iBAQ, and label-free quantification (LFQ) enabled. Carbamidomethylation was specified as fixed cysteine modification, and N-terminal acetylation, Met oxidation, and Thr-to-βES (+9.984 Da) were set as variable modifications. The MS/MS spectra were searched against the human UniProt database (UniProt Accession: UP000005640) including reverse peptide sequences for FDR estimation (downloaded June 2017). The used search database included reviewed and unreviewed human UniProt entries (159.743 in total). A maximum of two missed cleavages were allowed. Mass tolerance was set at 4.5 and 20 ppm for precursor ion and fragment ions, respectively. FDR was set at 0.01 for both peptide and protein levels. A minimum of two ratio counts were required for protein quantification.

After removing common contaminants and decoy database hits from the resulting MaxQuant proteinGroups file, alias gene names were replaced with official gene symbols using the Limma package (22). If this resulted in duplicate entries, the entry with the highest number of razor + unique peptides was retained. Protein groups were required to have at least two assigned peptides, of which at least one was a unique peptide. Differentially enriched protein analysis was performed using the DEP package (23). All protein groups that were detected in at least all but one replicate of at least one condition were considered for downstream analysis. Imputation of missing values was performed using the MinProb method with default settings. All proteins that showed an adjusted *p* value <0.05 and an absolute fold change >2 were considered to be differentially expressed. Supplemental Table S10 contains all results from protein identifications analysis of the proteome.

### Secretomics Sample Preparation, Enrichment of Nascent Protein, and On-Bead Digestion

Cleared supernatant samples were thawed and concentrated to 250 μl using 3 kDa centrifugal filters (Amicon) and 1× complete protease inhibitors (Roche) were added. The CuAAC reaction to enrich for βES-labeled proteins was set up following the Click-iT Protein Enrichment Kit (Invitrogen, C10416) manufacturer's instructions. Instead of the beads supplied with the Click-iT Protein Enrichment Kit, azide-functionalized agarose beads (Sigma-Aldrich, 900957) were used to enrich alkyne-labeled NSPs. Briefly, 100 μl of azide bead slurry was washed with 1 ml ultrapure water, after which 250 μl of concentrated medium, 250 μl of urea buffer, 500 μl of 2× catalyst solution, and 1× complete protease inhibitors were added. After 16 to 20 h incubation at RT with constant rotation, beads were washed with 1 ml ultrapure water. Next, reduction and alkylation of the bound proteins was done by incubating the beads with 10 mM DTT in 500 μl of SDS buffer for 15 min while shaking, followed by incubation with 50 mM iodoacetamide in 500 μl of SDS buffer for 30 min, shaking and protected from light. Beads were transferred to spin columns and washed with 20 ml of SDS buffer, 20 ml of 8 M urea in 100 mM Tris (pH 8), 20 ml 20% isopropanol, 20 ml 20% ACN (in MQ), and 5 ml of PBS. Bound proteins were digested through resuspension in 200 μl of freshly prepared digestion buffer (2 M urea, 100 mM Tris–HCl pH 8, 100 mM DTT) with 0.5 μg of mass spec-grade trypsin (Promega) and O/N incubation at RT while shaking. The digest was desalted and concentrated on C18 StageTips without acidification. Peptide labeling was done by dimethyl labeling (24), and StageTips were stored at 4 °C until measurement by LC-MS/MS.

### Secretomics LC-MS/MS Measurements and Data Analysis

Secretomic analysis was performed as previously described (25). In short, peptide samples were eluted from StageTips with elution buffer (80% ACN, 0.1% FA in ultrapure water), and light- and medium-labeled samples for the forward and reverse reactions were combined. Samples were reduced to 10% of the original volume by vacuum concentration and diluted in 0.1% FA to ∼12 μl. Sample (5 μl) was injected and peptides were separated using an Easy-nLC 1000 liquid chromatography system (Thermo Fisher Scientific) with a 44 min ACN gradient (7–30%), followed by washes at 60% and 95% ACN for a total of 60 min data collection. For the mass spectrometer settings see “Proteomics LC-MS/MS measurements and data analysis.” Subsequent protein identification and quantification was performed in MaxQuant v1.6.0.1 with standard settings and requantify enabled. Carbamidomethylation was specified as fixed cysteine modification, and *N*-terminal acetylation, Met oxidation, and Thr-to-βES (+9.984 Da) were set as variable modifications. Light (+0) and medium (+4) dimethyl labeling on the N termini and lysine residues was specified under “labels.” The MS/MS spectra were searched against the human UniProt database including reverse peptide sequences for FDR estimation (downloaded June 2017). Mass tolerance was set at 4.5 and 20 ppm for precursor ion and fragment ions, respectively. FDR was set at 0.01 for both peptide and protein levels. Protein quantification required two ratio counts.

For filtering method of MaxQuant protein groups, see “Proteomics LC-MS/MS measurements and data analysis.” Only proteins detected in both forward- and reverse-labeled samples were considered for downstream analysis. All proteins with a mean absolute fold change >2 in both the forward- and reverse-labeled experiment were considered to be differentially secreted. Supplemental Table S11 contains all results from protein identifications analysis of the secretome.

### GO and KEGG Pathway Enrichment Analysis of DEPs and NSPs

Gene ontology (GO) enrichment analysis including BP, cellular component (CC), and molecular function (MF) was performed by SRplot (https://www.bioinformatics.com.cn/en (accessed on: 22 November 2022)), and combined the clusterProfiler and pathview R package. For pathway enrichment the Kyoto Encyclopedia of Genes and Genomes (KEGG; https://www.genome.jp/kegg/) and SRplot was used (26, 27). For DEPs from single-shot proteomics, the average of three technical replicates was used as input, for NSPs from the secretome analysis the average of the forward and the reverse run was used.

### Omics Data Visualization

Heat maps were created using ComplexHeatmap (28) and other data visualizations were created with ggplot2 (29).

### Protease Cleavage Site Data Analysis

To interrogate potential protease cleavage sites in the collected proteomics data, mass spectrometry raw data files were matched to a human proteome database containing isoforms and contaminants (*Homo sapiens,* UniProt Accession: UP000005640, accessed January 2025, 83,385 protein entries) including 41,692 decoys = 50.0%) using FragPipe v.21.1 (MSFragger v.4.0) (30). Variable and fixed modifications for the proteome were set as above and cleavage specificity was set to “SEMI” and “stricttrypsin” (maximum 2 missed cleavages) to allow detection of nontryptic peptides. Mass tolerances for precursor and fragment ions were set to 20 ppm. An isotopic error was set to 2 Da. FDR was determined using Philosopher (v.5.1.0) and set at both the protein and peptides levels. LFQ for cellular proteome analysis was performed using IonQuant (v.1.10.12) (31). The resulting quantification values were then processed in Perseus (v.1.6.0.7) (32), where a log_2_ transformation was applied and quantifications filtered to contain a minimum three of three valid values in at least one of the two groups. The remaining missing values were imputed based on a normal distribution (σ-width = 0.3 and σ-downshift = 1.8) before a two-sample *t* test was applied to calculate fold change and *p* value. Data visualization was performed using R (v.4.3.1), where nontryptic peptides were also selected for by filtering for those which do not end with arginine (R) or lysine (K) and/or follow an R/K in the overall protein sequence. The resulting nontryptic (potential N or C termini) peptides were visualized by volcano plot using ggplot2. Supplemental Table S9 contains all results from the FragPipe analysis.

### HPLC Kynurenine Quantification

For kynurenine quantification, cDC2s were cultured in 100 μl custom made tryptophan (Trp)-free RPMI 1640 (Gibco) supplemented with 10 μM l-Trp (Merck), 2 mM UltraGlutamine (Lonza), 1% antibiotic-antimycotic (Thermo Fisher Scientific) and 2% HS, with 0, 1 μM, or 10 μM Epacadostat (INCB024360; Selleck Chemicals) and indicated stimuli (20 μg/ml poly I:C and 4 μg/ml R848, Invivogen) in a round-bottom 96-well plate. After 48 h, supernatants were collected, centrifuged at 1500 rpm for 5 min to remove any cells or cell debris, and stored at −20 °C until further analysis for l-kynurenine by HPLC (33). As internal standard for HPLC measurements, 100 μl of a 10 μM 3-nitro-l-tyrosine (Sigma-Aldrich) PBS solution was added to 100 μl supernatant (1:1 dilution). Prior to HPLC measurements, proteins were precipitated by addition of 25 μl 2 M trichloroacetic acid (Sigma-Aldrich, final concentration 0.4 M) and centrifugation of samples at 13,800*g* for 5 min. Subsequently, 150 μl supernatant was transferred to HPLC vials and measured on the autosampling HPLC device (Shimadzu) with XSelect Peptide CSHTM C18 column (130 Å, 3.5 μm, 4.6 mm × 100 mm, Waters). l-kynurenine standards (Sigma-Aldrich) were run to generate a standard curve, prior to running the experimental samples. Manual integration of each chromatographic peak area at 360 nm was performed to determine peak area size. After subtracting, the area levels measured for medium and medium with 50% BLM-CM from the experimental samples, the concentrations of l-kynurenine were calculated by extrapolation into the linear standard curve.

### LE28 (LGMN) and BMV109 (Cathepsin) Activity Labeling

For LE28 and BMV109 labeling in cultured cells, purified cDC2s (100,000 cells per condition) were cultured in round-bottom 96-well plates in X-VIVO 2% HS, X-VIVO 2% HS with 50% BLM-CM, or with 1 ng/ml IL-6 (Miltenyi), 10 ng/ml M-CSF (PeproTech), with or without 2 ng/ml prostaglandin E2 (PGE2, Pfizer). After 40 h, cDC2s were washed and incubated for 1 h at 37 °C with 1 μM BMV109 or 1 μM LE28 to label cysteine cathepsin or LGMN activity, respectively. Cells were harvested with ice-cold PBS and a fraction of the cells was analyzed by flow cytometry for Cy5 signal. The remaining cells were washed with PBS twice, centrifuged at 10,000 *g* for 1 min at RT, the supernatant was removed, and cells resuspended in 9 μl hypotonic lysis buffer (50 mM Pipes pH 7.4, 10 mM KCl, 5 mM MgCl, 4 mM DTT, 2 mM EDTA, 1% NP40). Lysates were incubated on ice for 15 min, followed by centrifugation at 21,130*g* for 15 min at 4 °C to pellet debris. Cleared lysate was collected and diluted with Laemmli's sample buffer (40% glycerol, Tris/HCl (0.2 M, pH 6.8), 8% SDS, 10% βME, 0.04% bromophenol blue). After denaturing samples for 5 min at 95 °C, samples were separated using SDS-PAGE and in-gel fluorescence was measured by scanning on an Amersham Typhoon 5. Total protein normalization was performed based on either Coomassie staining or after transfer to polyvinylidene difluoride membrane (Millipore) followed by Ponceau S staining (Thermo Fisher Scientific). Protein staining intensity was determined using ImageJ software (https://imagej.net/software/fiji/downloads) and corrected for background signal and total protein loading, prior to normalization against the medium control condition.

For LE28 and BMV109 labeling in HD, PBMCs were obtained as described under “human blood immune cells.” Immediately following isolation, a total of 3·10^6^ cells per donor were cultured in a concentration of 1·10^6^ cells/ml in X-VIVO (2% HS) with 1 μM BMV109, LE28, or left untreated, for 1 h at 37 °C in 1.5 ml Eppendorf tubes. After incubation, cells were collected and analyzed by flow cytometry as described under “flow cytometry.” For LE28 and BMV109 labeling in PBMCs from melanoma patients, previously isolated PBMCs (using Lymphoprep), and subsequently stored in liquid nitrogen, were thawed and labeled as described above. Where indicated, cells were preincubated with 5 μM FJD005 for 1 h at 37 °C.

### Tumor Tissue Processing After CUSA

Melanoma brain metastasis tissue was surgically removed using cavitron ultrasonic surgical aspirator (CUSA). CUSA material was filtered over a 30 μM cell strainer to separate tissue from suspension, tissue was cut into pieces of max 2 to 4 mm and subsequently dissociated using the Tumor Dissociation Kit (Miltenyi, 130-095-929) and the gentleMACS Octo Dissociator with Heaters according to the manufacturer's protocol for soft tumor types (gentleMACS Program 37C_h_TFK_1). After incubation, the cell suspension was applied to a 30 μM MACS SmartStrainer. Enrichment of immune cells was achieved by Lymphoprep isolation, followed by washing steps until the supernatant appeared clear. BMV109 and LE28 labeling was performed as described above for PBMCs.

### OLINK Analysis

Samples from 24 h serum-free CM from BLM cells were sent to OLINK for simultaneous analysis of 96 protein biomarkers using the Olink Target 96 Immuno-Oncology panel. The quality control was handled by Olink specialists. Scaled NPX values from proteins detected in the several dilutions of the BLM-CM samples were used to plot the bar charts in Graphpad Prism software (V8).

## Results

### An *In Vitro* Model to Simultaneously Characterize the Proteome and Secretome of DCs

To study changes in protein production and secretion during the transition of DC2s to ti-DC3s, we utilized a previously established *in vitro* model consisting of primary human DC2s cultured with tumor-CM from the melanoma cancer cell line BLM (BLM-CM) (4). Single-shot proteomics with LFQ was employed to assess differences in the intracellular proteome (Fig. 1*A*). We also aimed to analyze the secretome using THRONCAT, for which DCs need to be cultured with βES to allow incorporation of βES in NSPs (Fig. 1, *A* and *B*). Since simultaneous and accurate assessment of the secretome and proteome requires cell culture with βES without affecting the viability, phenotype, and development of DCs, we first assessed the feasibility of THRONCAT in cDC2s before progressing to proteomics experiments.

The use of THRONCAT to analyze NSPs has been established in bacteria, *Drosophila melanogaster,* and the mammalian cell lines Ramos and HeLa (18). We explored the feasibility of THRONCAT in cDC2s in X-VIVO medium (including HS), as this is an optimal culture medium for primary cells. cDC2s isolated from peripheral blood of HDs were cultured with increasing concentrations of βES. We quantified the incorporation of βES into the nascent proteome by flow cytometry through CuAAC of a sulfo-Cy5-azide to the alkyne in βES, after fixation and permeabilization of the cells (Fig. 1*B*) (18). After 1 h incubation with 0.4 mM, 1 mM, and 4 mM βES, we observed a dose-dependent increase in sulfo-Cy5 signal in cDC2s and control HeLa cells, indicating successful βES incorporation (Fig. 1*C*, Supplemental Fig. S1*A*). Herewith, we add DCs in X-VIVO medium to the conditions compatible with THRONCAT, further extending the applicability of this metabolic profiling technique.

To establish the time required for BLM-CM–driven conversion of cDC2s into ti-DC3s, we measured the conversion kinetics. Approximately 50% of DC2s acquired CD14 after 24 h, which increased to 70% after 40 h (Supplemental Fig. S1). In addition, we assessed the effects of metabolic labeling with βES on both conversion and viability (Supplemental Fig. S1). Toxic effects were limited when the βES labeling time did not exceed 12 h. This led to a final experimental set-up in which cDC2s were cultured in medium or BLM-CM for 28 h and labeled with 1 mM βES for an additional 12 h, followed by harvesting the cells for proteomic analysis (Fig. 1*D*). Using this timeline, the βES incorporation occurs while cDC2s are transitioning into ti-DC3, the majority of cDC2s will be ti-DC3, while minimizing toxic effects. Lastly, we used human monocytes, an easily obtained myeloid cell population, to obtain an indication of the number of detectable proteins achievable by THRONCAT in primary myeloid cells. Labeling for 12 h with βES followed by LC-MS/MS analysis identified 435 NSPs from 200 μg protein input, affirming that THRONCAT is suitable for use in human monocytes (Supplemental Fig. S2*A*, Supplemental Table S1).

### Proteomic Changes in cDC2s During Their Tumor-Induced Conversion to CD14^+^ DC3s

**Fig. 2:**
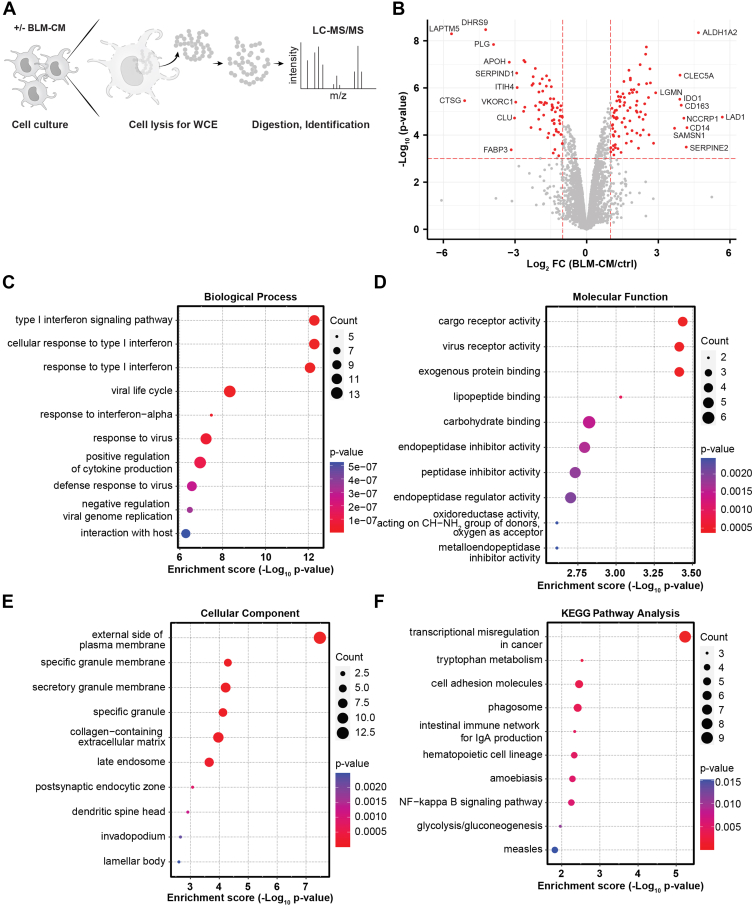
**Proteomics of cDC2s during tumor-induced conversion towards CD14^+^ DC3s.***A,* schematics showing approach for the proteome analysis of DC2s *versus* tumor-induced DC3s, generated using BLM-conditioned medium (BLM-CM). After the culture period, cells are lysed and proteins digested followed by LC-MS/MS analysis. *B,* volcano plot displaying the log_2_ fold change against −log_10_ statistical *p* value for all proteins detected in the LC-MS/MS analysis, performed in technical triplicates. Top 10 upregulated and downregulated proteins are labeled with their gene symbol. *Red dots* indicate significant DEPs with *p* adjusted <0.05 and |FC| > 1. Differentially upregulated proteins in BLM-CM conditions were subjected to gene ontology enrichment analysis for biological process (*C*), molecular function (*D*), cellular components (*E*), and KEGG pathway enrichment analysis (*F*), all performed by SRplot. Top 10 terms are displayed as bubble plots based on *p* value. N = 1, five individual donors pooled. See also Supplemental Figure S2. cDC2, type 2 conventional dendritic cell; KEGG, Kyoto Encyclopedia of Genes and Genomes; DEP, differentially expressed protein.

To acquire sufficient cell numbers for the proteomics analysis of cDC2s, we isolated cDC2s from apheresis material from a total of five HDs. After isolation, the cDC2 fractions (purity >94%, Supplemental Fig. S2*B*) of different donors were pooled and divided over the cell culture condition without BLM-CM (23.6·10^6^ cells) and with 50% BLM-CM (19.3·10^6^ cells). Cells were cultured for 40 h and harvested. The protein yield after cell lysis was 202 μg for the medium condition and 557 μg for the cDC2s cultured in BLM-CM (Supplemental Table S1). Analysis of the triplicate runs on LC-MS/MS showed low variation between technical replicates (Supplemental Fig. S2*C*). Of a total of 4715 proteins detected in the proteome analysis, 157 were DEPs (Log_2_ FC > 1, p_adjusted_ < 0.05) between medium (DC2s) and BLM-CM cultured (ti-DC3s) (Fig. 2, *A* and *B*, Supplemental Tables S2 and S10). Of these, 89 proteins were found upregulated in ti-DC3s and 68 in DC2s cultured in tumor-free conditions.

DEPs were subjected to GO analysis in terms of BP, MF, and CC. Proteins upregulated in ti-DC3s were mainly involved in type I interferon (IFN-I) signaling pathways and responses (BP) (Fig. 2*C*), cargo and virus receptor activity and exogenous protein binding for MF, and plasma and secretory granule membrane components (CC) (Fig. 2, *D* and *E*). The KEGG pathway analysis revealed the highest enrichment for transcriptional misregulation in cancer, Trp metabolism, and cell adhesion molecules (Fig. 2*F*). Specifically, the top 10 DEPs by fold change in ti-DC3s included CD14 and CD163, which are the phenotypic hallmarks of DC3s (2) and therefore strongly support the proteomic results (Fig. 2*B*, Table 1). The CD11b integrin (ITGAM), which is higher expressed on DC3s *versus* DC2s (4), was also found increased in the lysates of ti-DC3s (Log_2_ FC 1.83, p_adj_ = 2.17·10^−14^) (Supplemental Table S2). The DC2s residing in medium without any stimulation for the culture period did not display protein upregulation in any process or pathway strongly related to immune responses (Supplemental Fig. S2, *D*–*G*).

**Table 1:** Top 10 significantly upregulated proteins in ti-DC3s (BLM-CM) and DC2s (medium (ctrl))

Upregulated in BLM-CM	Gene symbol	Protein ID	Log_2_ FC (BLM-CM/ctrl)	*p* value adjusted	*p* value unadjusted
	*LAD1*	O00515	5.68	1.58E-07	1.74E-05
	*ALDH1A2*	O94788	4.68	2.17E-14	4.52E-09
	*CD14*	P08571	4.2	2.65E-05	4.87E-05
	*SERPINE2*	P07093	4.17	8.14E-03	3.25E-04
	*NCCRP1*	Q6ZVX7	4.07	2.77E-07	1.93E-05
	*CD163*	Q86VB7	3.96	3.49E-11	5.42E-06
	*CLEC5A*	Q9NY25	3.91	2.17E-14	2.87E-07
	*IDO1*	P14902	3.9	1.42E-13	3.03E-06
	*SAMSN1*	Q9NSI8	3.68	3.35E-05	5.16E-05
	*LGMN*	Q99538	2.89	2.17E-14	1.61E-06

Upregulated in medium	Gene symbol	Protein ID	Log_2_ FC (Ctrl/BLM-CM)	*p* value adjusted	*p* value unadjusted
	*LAPTM5*	Q13571	5.66	2.17E-14	5.05E-09
	*CTSG*	P08311	5.11	5.99E-13	3.48E-06
	*DHRS9*	Q9BPW9	4.23	2.17E-14	3.36E-09
	*PLG*	P00747	3.9	2.17E-14	1.44E-08
	*APOH*	P02749	3.24	2.17E-14	8.11E-08
	*FABP3*	P05413	3.16	1.37E-02	4.24E-04
	*CLU*	P10909	3.02	2.59E-07	1.90E-05
	*VKORC1*	Q9BQB6	2.97	2.37E-12	4.02E-06
	*SERPIND1*	P05546	2.93	2.17E-14	2.35E-07
	*ITIH4*	Q14624	2.91	2.17E-14	9.66E-07

CM, conditioned medium; FC, fold change; ti-DC3, tumor-induced DC3.

### Increased IDO and LGMN Activity in DC3s

**Fig. 3:**
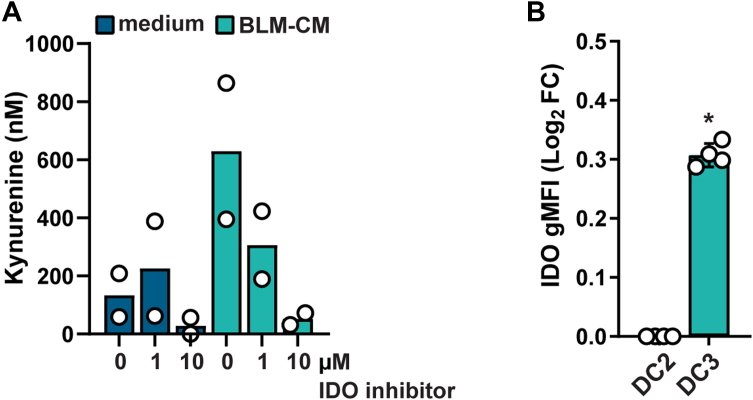
**Increased IDO activity in tumor-associated DC3s.***A,* kynurenine levels in 48 h supernatants of 50,000 dendritic cells (DCs) measured by HPLC, to analyze conversion of L-tryptophan to kynurenine as measure of indoleamine-2,3-dioxygenase (IDO) activity. DCs were cultured in 100 μl medium or medium with 50% BLM-CM, containing 10 mM L-tryptophan and increasing concentrations of the IDO inhibitor Epacadostat. Each *circle* represents an individual donor, *bars* represent mean values (N = 2 independent experiments, ratio paired *t* test between kynurenine levels of DC2 and ti-DC3, without inhibitor, results in *p* = 0.046). *B,* intracellular IDO levels of freshly isolated DCs from healthy donors, shown as log_2_ fold change (FC) of CD1c^+^CD14^+^ DC3 *versus* CD1c^+^CD14^-^ DC2s. Each *circle* represents an individual donor (N = 4), *bars* are mean ± SD (Paired *t* test on raw values, ∗*p* < 0.05). CM, conditioned medium; ti-DC3, tumor-induced DC3.

To explore upregulated proteins potentially associated with the observed immunosuppressive function of ti-DC3s, we investigated the DEPs IDO1 and *LGMN*. The enzyme IDO1 showed a 14.9-fold increase in ti-DC3s (BLM-CM) *versus* DC2s (medium) (log_2_ FC 3.9, p_adj_ = 1.42·10^−13^) (Table 1). IDO1 catalyzes the conversion of the essential amino acid Trp to kynurenine, which inhibits T cell proliferation. Furthermore, IDO1 can signal through β-catenin (CTNNB1) (34, 35), and kynurenine signals through the aryl hydrocarbon receptor (AhR) (36), both of which were detected in ti-DC3 lysates (Supplemental Fig. S2*H*). AhR signaling induces apoptosis of effector T cells and differentiation of regulatory T cells, thereby suppressing T cell immunity. To assess if the increased IDO1 levels in ti-DC3s translate to increased functional activity, we measured kynurenine levels in the medium of ti-DC3s and compared this to the medium of DC2s. Supernatant from DC2s cultured in medium contained almost no detectable kynurenine (<200 nM) (Fig. 3*A*). In contrast, kynurenine levels around 600 nM were detected for ti-DC3s and showed a dose-dependent decrease upon Epacadostat (IDO inhibitor) treatment (Fig. 3*A*). Additionally, we assessed the effect of stimulation with the Toll-like receptor ligands poly I:C + R848, known potent inducers of IDO activity in DC1s (37), on DC2s and ti-DC3s. Subset-specific differences in IDO activity have been observed for DC1s *versus* DC2s, with the latter showing minimal IDO activity (37, 38). Here, we observed similarly low activity for DC2s, but high kynurenine levels of up to 3000 nM for ti-DC3s upon stimulation, revealing another DC-subset difference (Supplemental Fig. S2*I*). Lastly, in accordance with IDO1 expression in ti-DC3s, intracellular IDO1 levels were increased in CD14^+^ DC3s compared to CD14^-^ DC2s in freshly isolated DCs from healthy individuals (Fig. 3*B*). Altogether, we validated the increased IDO1 activity in ti-DC3 which, through the catabolism of Trp, contributes to T cell anergy, apoptosis, and increased regulatory T cells, collectively suppressing the antitumor immune response (36, 39).

**Fig. 4:**
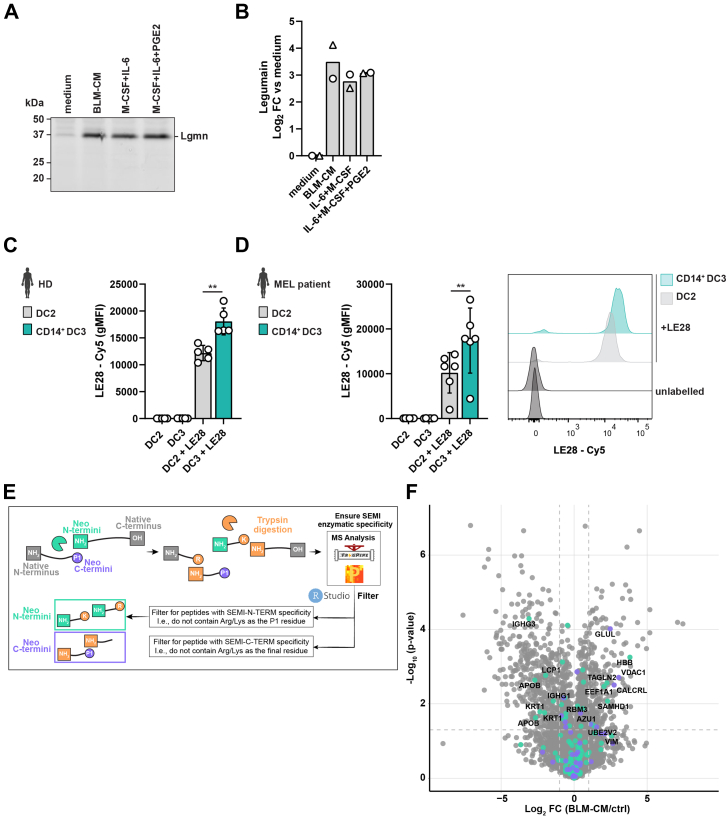
**CD14^+^ DC3s from healthy individuals and cancer patients have increased legumain activity.***A,* representative SDS-PAGE showing in-gel fluorescent signal of the activity-based probe LE28 for DCs after cultures in the depicted conditions. Protein signals were quantified and corrected for background signal in ImageJ, followed by total protein normalization based on Ponceau S staining. Log_2_ normalized fold change (FC) *versus* medium of the corrected signal intensities is displayed in (*B*). Each symbol depicts an individual donor (*bar* represents mean). See also Supplemental Figure S3. *C* and *D,* PBMCs from healthy donors (HDs) (*C*) or melanoma (MEL) patients (*D*) were labeled with the selective activity-dependent legumain probe LE28 for 1 h at 37 °C or left untreated, followed by analysis by flow cytometry. Each symbol shows geometric mean fluorescent intensity (gMFI) of a biological replicate with N = 5 for HD and N = 6 for MEL patients (mean ± SD, paired *t* test). *D,* right shows a representative histogram of LE28-Cy5 signal in depicted DC subsets. *E,* workflow to interrogate potential protease cleavage sites in LC-MS/MS data. Detection of nontryptic peptides was enabled by SEMI-cleavage specificity and filtering for those that do not end with arginine (R) or lysine (K) and/or follow an R/K in the overall protein sequence. *F,* volcano plot displaying the log_2_ fold change against −log_10_ statistical *p* value for all nontryptic peptides detected in the proteomics analysis. *Colored dots* indicate peptides with asparagine as previous amino acid (*green*) or as final amino acid (*purple*). Of these, significant DEPs with *p* adjusted <0.05 and |FC| > 1 are labeled with their gene symbol. ∗*p* < 0.05, ∗∗*p* < 0.01. See also Supplemental Figure S3. DC, dendritic cell; PBMC, peripheral blood mononuclear cell.

Asparaginyl endopeptidase, also known as *LGMN*, was increased 7.4-fold in ti-DC3s versus DC2s (log_2_ FC 2.89, p_adj_ = 2.17·10^−14^) (Table 1). In the tumor microenvironment, LGMN promotes tumor progression and is highly expressed by tumor-associated macrophages, while absent in classically activated macrophages (40). To assess whether ti-DC3s display higher LGMN activity correlating with increased protein abundance, we incubated cDC2s cultured in medium or BLM-CM with the quenched activity-based probe LE28, which becomes fluorescent upon covalently binding to active LGMN (41). We observed an 11.3-fold increase in LE28 signal (mean log_2_ FC 3.5), indicating a strong increase in LGMN activity in ti-DC3s (Fig. 4, *A* and *B*, Supplemental Fig. S3*, A* and *B*). Similar results were obtained with cDC2s stimulated with IL-6 + M-CSF (+PGE2), tumor-derived cytokines known to be responsible for the conversion of cDC2s to CD14^+^ DC3s (4, 42). To further investigate these findings in *in vivo–*developed DC3s, we labeled DC2s and DC3s isolated from HDs and melanoma patients and quantified the LE28 signal by flow cytometry (Supplemental Fig. S3*E*). In both cases, DC3s showed a significant increase in LGMN activity compared to DC2s, suggesting enhanced LGMN is DC3-intrinsic (Fig. 4, *C* and *D*, Supplemental Fig. S3, *C* and *D*). Of note, melanoma patients have increased DC3 frequencies (3, 4), thereby exacerbating the elevated LGMN levels compared to healthy individuals.

LGMN has a specific preference to cleave substrates after asparagine residues (43). This enables analysis of the proteomics data for potential peptides cleaved by LGMN based on cleavage site (44) (Fig. 4*E*). First, we selected semitryptic peptides to distinguish peptides arising from endogenously cleaved proteins from those cleaved by trypsin after K/R residues (Supplemental Fig. S3*F*, Supplemental Table S3). We observed 233 nontryptic peptides enriched in ti-DC3s, while 423 were enriched in cDC2s, reflecting significantly altered proteolytic networks in these cells. Among those enriched in ti-DC3s were the known cathepsin substrates CD74 and HLA (45), although it should be noted that cathepsin cleavage specificity is too broad to accurately assign cathepsins as the proteases responsible for these cleavages. To identify potential LGMN substrates, we filtered the nontryptic peptides for those arising after asparagine cleavage. Eleven peptides from eleven different proteins were enriched in ti-DC3s compared to seven peptides derived from four different proteins in DC2s (Fig. 4, *E* and *F*, Supplemental Table S4). Of these, transgelin (*TAGLN*) (44, 46), hemoglobin (*HBB*) (47), and voltage-dependent anion channel protein (48) have been previously described as LGMN substrates. Although it is possible that other proteases are responsible for this asparaginyl endopeptidase activity, this constraint significantly refines the pool of candidate LGMN substrates within these cells, which can be further validated in the future.

Further in line with the immunosuppressive characteristics of tumor-induced CD14^+^ cDC2s is the increased expression of aldehyde dehydrogenase 1 family, member A2 (ALDH1A2) (Table 1). ALDH1A2 is associated with the differentiation of DCs to tolerogenic DCs (49, 50, 51). It is the rate-limiting enzyme for retinoic acid in DCs, thus higher ALDH1A2 levels increases retinoic acid levels, which causes an expansion of regulatory T cells while suppressing Th1 and Th17 differentiation (49, 50, 51). Altogether, we examined changes in the proteome during tumor-induced conversion of DC2s to DC3s and demonstrate this is accompanied by an increase in functionally active IDO1 and LGMN.

### THRONCAT to Analyze the Secretome of ti-DC3s

**Fig. 5:**
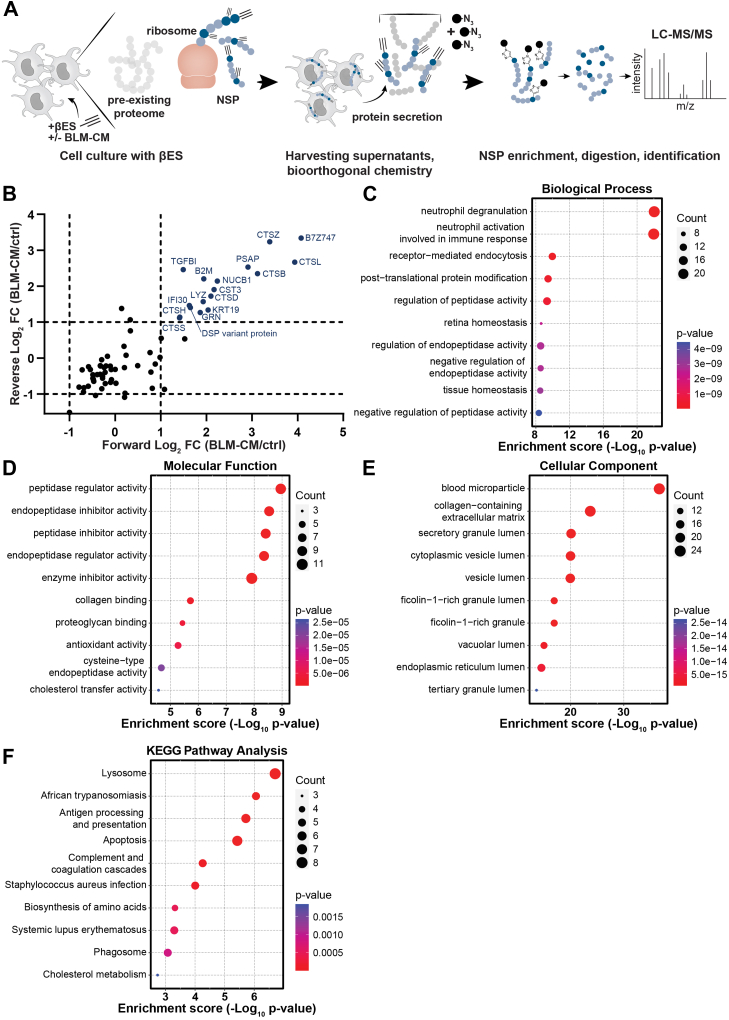
**THRONCAT to analyze the secretome of cDC2s and tumor-induced DC3s.***A,* schematics of THRONCAT workflow to analyze secreted newly synthesized proteins (NSPs) from cDC2s. cDC2s are labelled with βES during culture with or without BLM-CM, supernatant is collected, and NSPs selectively enriched using conjugation of the alkyne in βES to azide agarose beads. NSPs are digested and resulting peptides are subjected to LC-MS/MS. *B,* scatter dot plot showing detected NSPs in forward and reverse mass spectrometry run. Significant differentially expressed NSPs in both replicates (Log2 FC > 1, *p* < 0.001) are shown in *blue* and labeled with their gene symbol. All detected NSPs were subjected to gene ontology enrichment analysis containing (*C*) biological process, (*D*) molecular function, (*E*) cellular component, and (*F*) KEGG pathway enrichment analysis, performed by SRplot. Top 10 terms are displayed as bubble plots based on *p* value. See also Supplemental Figure S4. THRONCAT, threonine-derived noncanonical amino acid tagging; cDC2, type 2 conventional dendritic cell; βES, β-ethynylserine; CM, conditioned medium; KEGG, Kyoto Encyclopedia of Genes and Genomes.

We next employed THRONCAT to analyze the secretome of DC2s and ti-DC3s. To obtain insights into the proteins secreted by DC2s during their conversion into ti-DC3s, we collected supernatant from DC2s cultured for 28 h with BLM-CM, followed by a 12 h βES labeling period (Fig. 1*D*). After collecting the supernatant from cell cultures, NSPs were separated from the preexisting proteome and secretome by CuAAC of βES-containing proteins to azide agarose beads (Fig. 5*A*). Subsequent elution, digestion, and LC-MS/MS analysis identified a total of 66 secreted NSPs (Supplemental Table S5). Of these, 17 were significantly increased in the supernatant of ti-DC3s compared to DC2s in tumor-free culture conditions (FC > 2 in forward and reverse run) (Fig. 5*B*). Overall, the 66 detected NSPs were mainly involved in release of granules (BP neutrophil degranulation) and (endo)peptidase regulator and inhibitor activity (MF) (Fig. 5, *C* and *D*). For the CCs, microparticles, ECM, and several granule and vesicle lumens were enriched (Fig. 5*E*). Enriched pathways according to the KEGG database are lysosome, antigen processing and presentation, and apoptosis (Fig. 5*F*).

The THRONCAT secretome analysis revealed increased transforming growth factor-β–induced protein (TGFβI (Log_2_ FC 1.98)) in ti-DC3s. As the name implicates, TGFβI is downstream of the TGFβ signaling cascade (52). We then determined the TGFβ levels in BLM-CM using OLINK (OLINK Target 96 Immuno-Oncology Panel) and found increased levels of TGFβ compared to controls (Supplemental Fig. S4*A*), potentially inducing the increase in TGFβI observed in ti-DC3s. We also investigated the detected beta-2 microglobulin (B2M), a component of major histocompatibility complex class I molecules and frequently found elevated in blood of cancer patients (53). Analysis of DC2s and DC3s from melanoma patients for surface expression of B2M showed a strong increase in B2M expression on DC3s, thereby supporting our *in vitro* findings using *in vivo* developed ti-DC3s (Supplemental Fig. S4*B*). Taken together, we demonstrate for the first time that THRONCAT can track secreted NSPs of human DCs with which we found increased TGFβI and B2M secretion by DC3s.

### Tumor-Associated DC3s have High Levels of Intracellular Cathepsin Activity

**Fig. 6:**
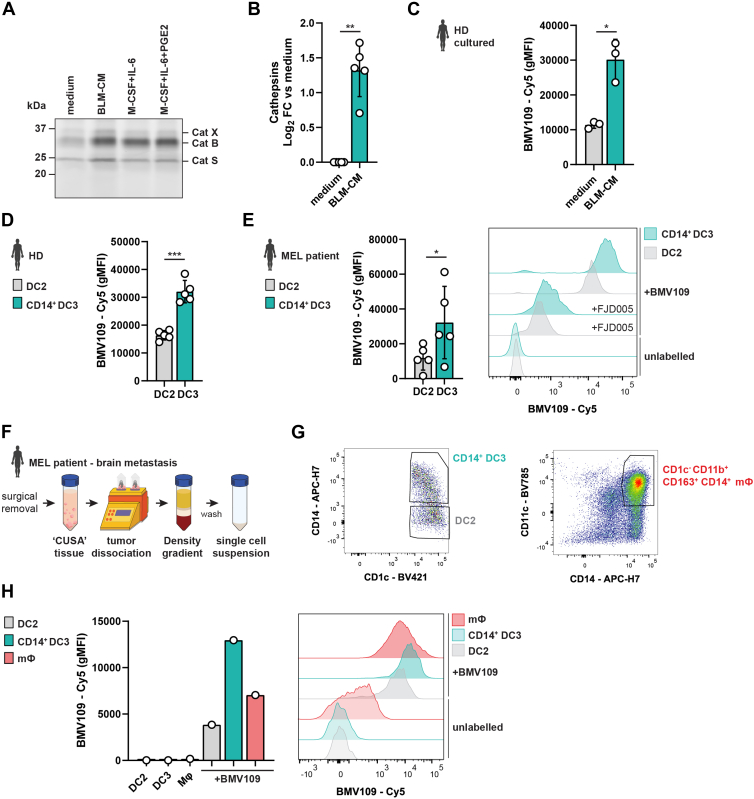
**Intracellular cathepsin activity in *in vitro* tumor-induced and *in vivo* tumor-derived DC3s.***A,* representative SDS-PAGE showing in-gel fluorescent signal of the activity-based probe BMV109 for DC2s from healthy donors (HDs) after culture with BLM-CM, M-CSF + IL-6, or M-CSF + IL-6 + PGE2. *B,* After BMV109 labeling, protein signals combined for all detected cathepsins, were quantified and corrected for background signal in ImageJ, followed by total protein normalization (Ponceau S and Coomassie staining, Supplemental Fig. S5, *A* and *B*). Corrected signal intensities were used to calculate log_2_ normalized fold change (FC) *versus* medium (N = 5 biological replicates, mean ± SD, paired *t* test). *C,* fractions of the DC2 samples shown in (*B*) were acquired by flow cytometry to quantify the BMV109-Cy5 signal. N = 3 biological replicates, mean ± SD, paired *t* test. *D* and *E,* PBMCs from HDs (*D*) or melanoma (MEL) patients (*E*) were labeled with BMV109 for 1 h at 37 °C with or without cathepsin inhibitor FJD005, followed by analysis by flow cytometry. Each symbol shows geometric mean fluorescent intensity (gMFI) of a biological replicate with N = 5 for HD and MEL patients (mean ± SD, paired *t* test). *E, right* shows a representative histogram of BMV109-Cy5 signal in depicted DC subsets. *F–H,* melanoma brain metastasis sample was processed as depicted in (*G*), followed by labeling with BMV109 for 1 h at 37 °C or left untreated. Cells were analyzed by flowcytometry and gated for DC2, CD14^+^ DC3, and macrophages (mɸ) as shown in (*H*), with their BMV109-Cy5 gMFI depicted in (*I*) in a bar chart (*left*) and histogram (*right*). ∗*p* < 0.05, ∗∗*p* < 0.01, ∗∗∗*p* < 0.001, and ∗∗∗∗*p* < 0.0001. See also Supplemental Figure S5. PGE2, prostaglandin E2; PBMC, peripheral blood mononuclear cell; CM, conditioned medium; M-CSF, macrophage colony-stimulating factor; IL, interleukin; DC, dendritic cell.

Of the proteins that were significantly increased in the NSP secretome in ti-DC3s, six belong to the cathepsin family (Fig. 5*B*). For instance, cathepsin L was found significantly upregulated in both the lysate (FC 2.36) and secretome (FC 9.85), indicating an increase in synthesis and secretion. To examine intracellular cysteine cathepsin activity, we labeled DC2s and ti-DC3s with fluorescent quenched activity-based probe BMV109. This probe interacts in an activity-dependent fashion to covalently label cysteine cathepsins (54). DC2s were first cultured with BLM-CM or a combination of IL-6, M-CSF, and PGE2 for 40 h. Labeling of intact DCs with BMV109 was followed by hypotonic lysis and analysis by fluorescent SDS-PAGE. This showed increased BMV109 labeling (FC ∼2.3) of cathepsin X, B, and S in ti-DC3s (Fig. 6, *A* and *B*, Supplemental Fig. S5, *A–C*). In addition to analysis by fluorescent SDS-PAGE, which enables separation of the different cathepsins, we evaluated the BMV109 signal in a fraction of the same samples by flow cytometry. As expected, ti-DC3s exhibited a strong increase in BMV109 signal compared to DC2s, while labeling was completely blocked by the cathepsin inhibitor FJD005 (55) (Fig. 6*C*, Supplemental Fig. S5*D*). The comparable results between SDS-PAGE and flow cytometry confirmed that analysis of BMV109 signal by flow cytometry can be utilized to assess cysteine cathepsin activity in DCs. This enabled us to investigate cysteine cathepsin activity in DC2s and DC3s with limited cell numbers, as is the case for DCs derived from peripheral blood of HDs and melanoma patients. In both HDs and melanoma patients, DC3s contain increased cathepsin activity compared to DC2s (Fig. 6, *D–E*, Supplemental Fig. S5*E*). Next, we extended this analysis to DC3s directly isolated from tumor tissue of a melanoma patient. After the melanoma brain metastasis was surgically removed using CUSA ablation, the tumor tissue was dissociated, tumor cells removed, and the remaining immune cells were labeled using BMV109 (Fig. 6*F*). Interestingly, cathepsin activity of DC3s not only surpassed that of DC2s but also that of tumor-associated CD11c^+^ macrophages (Fig. 6, *G–H*, Supplemental Fig. S5*F*) which are known to have high expression of cysteine cathepsins (55). Altogether, we identified high levels of cysteine cathepsin activity as a characteristic feature of ti-DC3s.

## Discussion

Proteome and secretome analyses of DCs are essential to obtain a deeper understanding of cell type–specific BPs, such as those involved in environmentally induced conversions or cell states. In this work, we characterized the proteome and nascent secretome of DC2s during their tumor-induced conversion to immune incompetent CD14^+^ DC3s (4). We conducted a proteome analysis of DC2s in tumor-free conditions compared to DC2s cultured with melanoma-secreted factors, revealing increased IDO1 and LGMN in ti-DC3s. In addition, we demonstrated for the first time the metabolic labeling technique THRONCAT in human DCs, to label NSPs with a bioorthogonal Thr analog. We applied THRONCAT to investigate the newly synthesized secretome and detected increased levels of cathepsins and TGFβI in DC3s. Together, we characterized the baseline and melanoma-induced proteome and secretome in DCs, providing new insights in protein-driven mechanisms characteristic of tumor-associated DC3s and able to supports tumor malignancy.

Previous studies reporting quantitative proteomic data of myeloid cells mostly studied changes in the proteome of monocyte-derived DCs (56, 57, 58). However, studies on primary human DC subsets (pDC, cDC1, DC2, DC3) have so far been limited (59, 60, 61). Proteomics analysis on freshly isolated CD1c^+^ myeloid DCs was performed by Schlatzer *et al.* (60)*,* while Worah *et al.* integrated proteome and transcriptome data of pDCs, cDC1s, and cDC2s, to generate signatures for each DC subset, except for DC3, which was yet to be identified (61). Through quantitative single-shot proteomics, we report the first proteomic characterization of ti-DC3s *versus* DC2s cultured in tumor-free conditions, detecting 157 DEPs. The GO enrichment analysis of DEPs upregulated in ti-DC3s showed strong enrichment for IFN-I signaling and responses. Complementary to our findings, Girard *et al.* reported the activation of an NF-κB-driven maturation program by IFN-β in DC3s (defined as CD5^-^CD1c^+^CD14^+/−^) and shared with cDC2s (62). Although this was not studied in ti-DC3s, the finding of an IFN-I–induced maturation program in DC3s and cDC2s provides a possible purpose for the upregulated IFN-I responses observed here.

Among the top 10 upregulated DEPs in tumor conditions were the enzymes IDO1 and LGMN. We assessed these two proteins in more detail in ti-DC3s, because of their known immunosuppressive and protumor effects. IDO1 catalyzes Trp to kynurenine, which drives impaired T cell proliferation, promotes regulatory T cell expansion, and tumor progression (36, 39, 63, 64). We confirmed *in vitro* that the increase in IDO1 expression correlated with increased kynurenine levels in the medium of ti-DC3s. Accumulating Trp metabolites, such as kynurenine, bind the AhR which induces the differentiation of regulatory T cells and the apoptosis of effector T cells, further enhancing the immune inhibitory effect on the T cell compartment (36, 64, 65). Hereby, IDO1 creates a protumor, immunosuppressive environment that accelerates tumor growth and resistance to immunotherapy (36, 64, 65, 66). Therefore, the increased IDO1 and kynurenine levels reveal a pathway that contributes to the immunosuppressive phenotype of ti-DC3s. When considering why ti-DC3 display enhanced IDO1, an IL-6-dependent STAT3 signaling loop is a likely candidate. AhR stimulates IL-6–dependent STAT3 signaling, which maintains IDO1 expression and thereby immunosuppression in several human cancers (64). IL-6 is also the driving factor for STAT3-dependent expression of IDO1 in monocyte-derived DCs (63). Our previous findings show that IL-6 is present in high amounts in BLM-CM and is the main driver of DC2 to ti-DC3 conversion (4). The clinical relevance of the IDO1-AHR-IL6-STAT3 transcriptional circuit is underscored by the associations of high IDO1 expression with reduced relapse-free survival in lung carcinoma patients (64). Besides IL-6, cancer cell–secreted Wnt5a induces IDO1 expression through β-catenin (CTNNB1) activation in DCs (34, 35). We observed an almost 2-fold increase of CTNNB1 in ti-DC3s compared to DC2s. In summary, increased IDO1 is an immunosuppressive characteristic of ti-DC3s that could be driven by increased IL-6 and CTNNB1 signaling.

LGMN, the asparaginyl endopeptidase, drives tumor progression by immunosuppressive polarization of macrophages, inhibition of anti-tumor T cell responses, and promoting tumor migration and invasion (67, 68, 69, 70). High LGMN expression colocalizes with tumor-associated macrophages in the tumor microenvironment and correlates with poor prognosis (71, 72, 73). Here, we add ti-DC3 to the population of cells that have high LGMN expression. Considering the described protumor and immunosuppressive effect of LGMN, the detected increase in ti-DC3 compared to DC2s represent a mechanism that ti-DC3s could employ to suppress the antitumor response and support tumor progression.

Ladinin-1 (LAD1) was the most strongly upregulated protein in ti-DC3s. LAD-1 has been associated with activation of the EGF-to-ERK pathway in tumor cells (74, 75). Although it has been shown that ERK activation induces tolerogenic DCs and expands regulatory T cells (76), whether LAD-1 is involved in regulation the ERK activation in DCs as well has not yet been studied. SerpinE2 (SERPINE2), which was also highly upregulated in ti-DC3s, is likewise involved in ERK regulation (77). We also detected SAM domain, SH3 domain, and nuclear localization signals 1 gene (SAMSN1 alias HACS1) upregulated in ti-DC3s. Although HACS1 is not well-characterized in human DCs, *Hacs1*^−/−^ mice displayed enhanced immunity suggesting an immunoinhibitory role for HACS1 (78). Finally, concordant with the increased PD-L1 expression on DC3s compared to DC2s, we found the PD-L1 protein regulator CMTM6 (79, 80) upregulated in ti-DC3s. In summary, we characterized tumor-induced changes in the DC2 proteome of which several are associated with immunosuppression and protumor effects, including IDO1 and LGMN, through which melanoma-induced DC3s could impair antitumor immunity.

To supplement the cellular proteome analysis, we performed a secretome analysis of ti-DC3s with THRONCAT. THRONCAT facilitates the LC-MS/MS identification of secreted NSPs without the need for *a priori* minimization of non-DC–derived proteins in media, or complicated elimination procedures during sample preparation. As demonstrated by Ignacio *et al.* in the first THRONCAT study, THRONCAT detects ∼7 times more proteins in βES-labeled conditions (enriched for NSPs) compared to nonlabeled conditions (background or preexisting proteins), indicating only approximately 14% of proteins might not be a NSP (18). Subsequent subtractions of these detected background proteins from the protein hits in βES-labeled conditions provides a dataset of highly enriched NSPs with minimal uncertainties about peptide origin. This overcomes hurdles encountered in previous proteome analyses, such as the study by Ghanem *et al.*, where approximately 94% of detected peptides corresponded to media additives, and some were thought to be derived from cellular contaminations (59, 81). Of note, a limitation of our study is that we were not able to include a nonlabeled control (due to limited cell numbers of this low abundant DC subset) or use SILAC-labeling, as the required starvation affects DC behavior. Consequently, we cannot rule out that a minority of the detected proteins are interfering preexisting proteins instead of NSPs. Filtering the analysis for βES-labeled peptides is not feasible, as βES stays covalently bound to the azide-functionalized beads when peptides are cleaved off during the sample preparation. However, the previously reported validations of THRONCAT (18) combined with our follow-up validation experiments of the main hits mitigate the impact of these limitations. Future studies should either include a nonlabeling condition where possible, to detect background proteins, or use SILAC-labels as an alternative when working with less sensitive cells that withstand medium starvation (24).

In our secretome analysis, we detected 17 DEPs for ti-DC3s, including TGFβI. TGFβI, also known as βig-h3 or keratoepithelin, localizes to the ECM for its function in ECM remodeling under physiological conditions (82). In cancer, TGFβI has a crucial role in enhancing tumor growth, tumor invasive potential, and immunosuppression (82, 83, 84, 85). Its expression is induced by TGFβ (52), which we detected in our analysis of BLM-CM. Moreover, high levels of secreted TGFβI are characteristic for tumor-associated macrophages, where it promotes cancer progression (84), which is in line with the macrophage-like phenotype of ti-DC3s. Furthermore, we detected increased secreted granulin, which derives from proteolytically cleaved progranulin. Progranulin induces expansion of regulatory T cell and inhibits T cell proliferation (86, 87). High expression of progranulin is mainly reported for monocyte-derived cells, where it is regulated by all-trans-retinoic acid, in line with our finding of upregulated ALDH1A2 in cDC2 lysates (88).

Of all identified DEPs in the ti-DC3 secretome, six belong to the cathepsin family. Cathepsins are mainly found in endo/lysosomal compartments and can be translocated to the extracellular space by alternative sorting from the Golgi or via lysosomal exocytosis, which can be induced by cellular stress, among others (45, 89). However, there is no increased toxicity for BLM-CM cultures compared to control conditions (both treated with βES), thereby excluding this as cause for the observed enrichment of cathepsins. Under physiological conditions, cathepsins in the extracellular space perform ECM remodeling. In pathophysiological conditions such as cancer, enhanced cathepsin secretion leads to aberrant ECM dynamics, which facilitates among others uncontrolled cell proliferation, migration, and tumor invasion (45). To assess the functional activity of the cathepsins, we labeled melanoma brain tumor-derived DC2s, DC3s, and macrophages with quenched activity-based probe BMV109. We demonstrated that tumor-associated DC3s have, in addition to increased cathepsin expression, the highest cysteine cathepsin activity of all cell types analyzed. Cathepsin activity in macrophages has been found to exert several pro-tumor effects including promoting angiogenesis, tumor growth, and invasion (90, 91, 92). It is not surprising that these cathepsin-related functions so far only have been attributed to macrophages, given the phenotypic similarities between ti-DC3 and tumor-associated macrophages (e.g. CD163, CD14, CD11b expression). However, our data reveal that ti-DC3s exhibit high levels of active cathepsins, suggesting a potential mechanism by which ti-DC3s, akin to macrophages, contribute to tumor progression.

In conclusion, we utilized proteomics and THRONCAT to characterize the tumor-induced conversion of DC2s to immune-impaired ti-DC3s. The obtained proteome and secretome datasets provide novel insights in tumor-induced BPs and in functional mechanisms employed by ti-DC3s. Thereby, we demonstrated the feasibility and applicability of THRONCAT in human immune cells. Labeling NSPs with THRONCAT in DCs holds great potential for future analyses of proteomic and secretomic changes to study protein-driven processes.

## Data Availability

The mass spectrometry proteomics data have been deposited to the ProteomeXchange Consortium *via* the PRIDE (93) partner repository with the dataset identifier PXD058584.

## Supplemental Data

This article contains supplemental data (34, 35, 62).

## Conflict of Interests

The authors declare no competing interests.

## Supplemental Data

Supplemental Table S11

**Abbreviations**
βES, β-ethynylserine
ACN, acetonitrile
AhR, aryl hydrocarbon receptor
ALDH1A2, aldehyde dehydrogenase 1 family, member A2
B2M, beta-2 microglobulin
BONCAT, bioorthogonal noncanonical amino acid tagging
BP, biological process
CC, cellular component
cDC, conventional dendritic cell
CM, conditioned medium
CuAAC, copper-catalyzed azide-alkyne cycloaddition
CUSA, cavitron ultrasonic surgical aspirator
DC, dendritic cell
DEP, differentially expressed protein
FA, formic acid
FDR, false discovery rate
GO, gene ontology
HD, healthy donor
HS, human serum
IDO1, indoleamine-2,3-dioxygenase 1
IFN-I, type I interferon
IL, interleukin
KEGG, Kyoto Encyclopedia of Genes and Genomes
LFQ, label-free quantification
LGMN, legumain
M-CSF, macrophage colony-stimulating factor
MF, molecular function
NSP, newly synthesized protein
PBMC, peripheral blood mononuclear cell
PD-L1, programmed death ligand 1
PGE2, prostaglandin E2
RT, room temperature
TGFβI, transforming growth factor-β–induced protein
THRONCAT, threonine-derived noncanonical amino acid tagging
ti-DC3, tumor-induced DC3
