# Supplementary material for: Proteome and Secretome Profiling of the Melanoma-Induced Transition Toward Immune Incompetent Dendritic Cells Reveals Enhanced IDO1, Cathepsin, and Legumain Activity
Source: Mol Cell Proteomics. 2025 Aug 9;24(9):101048. doi: 10.1016/j.mcpro.2025.101048 (PMC12454896; doi:10.1016/j.mcpro.2025.101048)
Supplement: Supplemental material [file mmc1.pdf]

Supplemental information

Supplementary tables and figures for:

Proteome and secretome profiling of the melanoma-induced transition towards immune incompetent dendritic cells reveals enhanced IDO1, cathepsin, and legumain activity

Anouk M.D. Becker[1,5], Bob J. Ignacio[2], Jelmer J. Dijkstra[3,4], Alexander R. Ziegler[5], Iván Ramos-Tomillero[1], Floris J. van Dalen[1,6], Laura E. Edgington-Mitchell[5], Michiel Vermeulen[3,4], Kimberly M. Bonger[2,7,8], I. Jolanda M. de Vries[1], Martijn Verdoes[1,7,9,#]

Corresponding author: Martijn Verdoes, m.verdoes1@lumc.nl

The file includes Tables S1, S2, S4-S8, Figures S1-S5. Table S3, Table S9-11 are attached as separate file.

Table S1. Cell numbers and corresponding protein yields in whole-cell protein extracts (WCE) of primary human myeloid cells

**Monocytes**				
**Culture condition:**	**1mM βES**	**4mM βES**		
Cell count - start culture	$15\cdot10^6$	$30\cdot10^6$		
Cell count - end culture	$7.9\cdot10^6$	$13.2\cdot10^6$		
**Culture condition:**	**1mM βES**	**4mM βES**	**4mM βES**	**4mM βES**
Cell count - input for WCE	$7.9\cdot10^6$	$7.9\cdot10^6$	$3.9\cdot10^6$	$1.4\cdot10^6$
Protein in lysates (µg)	377.7	316.8	152.6	102.4
**Dendritic cells**				
**Culture condition:**	**Medium**	**BLM-CM**	**(1mM βES)**	
Cell count - start culture	$23.6\cdot10^6$	$19.3\cdot10^6$		
Cell count - end culture	$4.68\cdot10^6$	$6.42\cdot10^6$		
Protein in lysates (µg)	202	557		

Table S2. Significantly upregulated proteins in cDC2s cultured in BLM-CM and control medium (ctrl), identified by mass spectrometry

Upregulated in BLM-CM	Gene symbol	Protein ID	Log_2 FC (BLM-CM/ctrl)	P-value adjusted	P-value unadjusted
	LAD1	O00515	5.68	1.58E-07	1.74E-05
	ALDH1A2	O94788	4.68	2.17E-14	4.52E-09
	CD14	P08571	4.2	2.65E-05	4.87E-05
	SERPINE2	P07093	4.17	0.00814	3.25E-04
	NCCRP1	Q6ZVX7	4.07	2.77E-07	1.93E-05
	CD163	Q86VB7	3.96	3.49E-11	5.42E-06
	CLEC5A	Q9NY25	3.91	2.17E-14	2.87E-07
	IDO1	P14902	3.9	1.42E-13	3.03E-06
	SAMSN1	Q9NSI8	3.68	3.35E-05	5.16E-05
	LGMN	Q99538	2.89	2.17E-14	1.61E-06
	SERPINE1	P05121	2.8	0.00357	2.24E-04
	SIGLEC1	Q9BZZ2	2.67	2.17E-14	6.13E-07
	CD40	P25942	2.65	2.17E-14	1.55E-07
	IFIT2	P09913	2.61	0.000348	9.81E-05
	CCDC50	Q8IVM0	2.59	2.17E-14	2.56E-06
	TRAF1	Q13077	2.51	2.17E-14	6.55E-07
	MSR1	P21757	2.5	2.17E-14	1.83E-08
	FSCN1	Q16658	2.47	2.17E-14	3.70E-08
	LAMP3	Q9UQV4	2.46	3.43E-08	1.36E-05
	TIMP1	P01033	2.43	1.06E-11	4.73E-06
	RELB	Q01201	2.37	6.27E-05	6.08E-05
	CKAP4	Q07065	2.36	5.22E-07	2.16E-05
	B7Z5V6	B7Z5V6	2.26	5.65E-09	1.04E-05
	FABP4	P15090	2.25	0.00039	1.02E-04
	NUB1	Q9Y5A7	2.24	2.17E-14	1.03E-07
	AGRN	O00468	2.2	1.49E-07	1.72E-05
	NDE1	Q9NXR1	2.18	2.17E-14	7.14E-07
	SEMA6B	Q9H3T3	2.17	0.0102	3.63E-04
	AOC1	P19801	2.11	7.14E-09	1.08E-05
	IRF4	Q15306	2.11	8.81E-13	3.63E-06
	IL7R	P16871	2.1	2.17E-14	1.50E-06
	MARCKSL1	P49006	2.1	0.000199	8.30E-05

CCR7	P32248	2.08	2.98E-09	9.50E-06
SDF4	Q9BRK5	2.08	3.64E-11	5.45E-06
GLUL	P15104	1.98	2.17E-14	1.19E-06
CD1B	P29016	1.92	2.17E-14	1.57E-06
CKB	P12277	1.9	8.08E-05	6.49E-05
CDK12	Q9NYV4	1.87	2.17E-14	1.20E-06
TRIP10	Q15642	1.87	0.00221	1.85E-04
ITGAM/CD11b	P11215	1.83	2.17E-14	3.84E-07
TGM2	P21980	1.83	2.17E-14	4.56E-07
MARCKS	P29966	1.82	2.62E-10	6.91E-06
MMP9	P14780	1.81	1.18E-06	2.51E-05
ISG20	Q96AZ6	1.79	2.17E-14	1.70E-06
IFIT3	O14879	1.77	2.17E-14	1.78E-07
IL4I1	Q96RQ9	1.72	2.36E-09	9.20E-06
NR4A3	Q92570	1.7	0.000248	8.85E-05
CMTM6	Q9NX76	1.66	0.00347	2.21E-04
PALM2AKAP2	C9JA33	1.62	2.07E-05	4.58E-05
IFI44	Q8TCB0	1.59	3.27E-12	4.17E-06
RAB9A	P51151	1.58	2.17E-14	2.54E-06
CRLF2	Q9HC73	1.57	3.03E-07	1.96E-05
ARMC9	Q7Z3E5	1.56	3.82E-11	5.48E-06
LILRB2	Q8N423	1.56	2.17E-14	5.31E-07
IFIT1	P09914	1.52	2.17E-14	1.77E-06
IL1RN	P18510	1.48	2.57E-06	2.92E-05
ETV3	P41162	1.44	0.0221	5.48E-04
FBP1	P09467	1.43	2.17E-14	2.38E-06
SERPINB9	P50453	1.4	1.48E-12	3.82E-06
H6PD	O95479	1.39	8.82E-11	6.05E-06
CYRIA	Q9H0Q0	1.37	2.98E-13	3.26E-06
TRAFD1	O14545	1.35	0.000553	1.14E-04
RFTN1	Q14699	1.33	2.17E-14	2.31E-06
TMED8	Q6PL24	1.31	0.0111	3.79E-04
GALM	Q96C23	1.28	2.17E-14	1.46E-06
ITGB7	P26010	1.28	2.64E-07	1.91E-05
CD86	P42081	1.26	1.88E-07	1.79E-05
MX1	P20591	1.26	7.95E-13	3.58E-06
HK3	P52790	1.25	1.74E-09	8.81E-06
CTSL	P07711	1.24	3.98E-07	2.06E-05
BCL3	P20749	1.22	2.27E-07	1.85E-05
COX17	Q14061	1.2	0.00911	3.43E-04
IFITM3	Q01628	1.2	0.00484	2.56E-04
C15orf48	A0A024R5U4	1.19	2.23E-09	9.12E-06
OAS1	P00973	1.19	0.000115	7.13E-05
MX2	P20592	1.15	1.98E-07	1.81E-05
REL	Q04864	1.15	8.91E-09	1.11E-05
XAF1	Q6GPH4	1.14	0.0335	7.01E-04
DAP	P51397	1.13	0.000518	1.11E-04
PBXIP1	Q96AQ6	1.12	0.00608	2.83E-04
LXN	Q9BS40	1.11	0.000353	9.85E-05
MATK	P42679	1.11	0.0134	4.19E-04
ISG15	P05161	1.1	8.45E-08	1.56E-05
ANTXR2	P58335	1.09	3.70E-05	5.29E-05
NRP2	O60462	1.09	7.34E-06	3.62E-05
PLAUR	Q03405	1.07	1.04E-05	3.91E-05
MRC1	P22897	1.06	4.39E-10	7.37E-06
CD83	Q01151	1.01	0.0092	3.45E-04
MTRNR2L1	P0CJ68	1.01	0.0203	5.22E-04

Upregulated in medium	Gene symbol	Protein ID	Log_2 FC (ctrl/BLM-CM)	P-value adjusted	P-value unadjusted
	LAPTM5	Q13571	5.66	2.17E-14	5.05E-09
	CTSG	P08311	5.11	5.99E-13	3.48E-06
	DHRS9	Q9BPW9	4.23	2.17E-14	3.36E-09
	PLG	P00747	3.9	2.17E-14	1.44E-08
	APOH	P02749	3.24	2.17E-14	8.11E-08
	FABP3	P05413	3.16	0.0137	4.24E-04
	CLU	P10909	3.02	2.59E-07	1.90E-05
	VKORC1	Q9BQB6	2.97	2.37E-12	4.02E-06
	SERPIND1	P05546	2.93	2.17E-14	2.35E-07
	ITIH4	Q14624	2.91	2.17E-14	9.66E-07
	CRACR2A	Q9BSW2	2.64	3.88E-09	9.86E-06
	APOB	P04114	2.63	2.17E-14	6.80E-08
	TMEM59	Q9BXS4	2.59	1.80E-10	6.59E-06
	ELANE	P08246	2.58	2.17E-14	7.88E-08
	A1BG	P04217	2.33	2.55E-05	4.83E-05
	CHI3L1	P36222	2.29	2.17E-14	7.70E-07
	APOA1	P02647	2.24	2.17E-14	3.34E-07
	PLD3	Q8IV08	2.24	2.17E-14	6.60E-07
	HP	P00738	2.15	2.17E-14	1.49E-07
	APOA2	P02652	2.14	5.88E-11	5.76E-06
	UCHL1	P09936	2.01	4.78E-07	2.13E-05
	AZU1	P20160	2	2.17E-14	1.50E-06
	SLC38A2	Q96QD8	2	1.37E-09	8.53E-06
	C4B	P0C0L5	1.96	2.51E-12	4.04E-06
	VTN	P04004	1.96	2.17E-14	6.60E-07
	ME1	P48163	1.95	5.00E-05	5.72E-05
	SCIN	Q9Y6U3	1.9	3.33E-12	4.18E-06
	APOD	P05090	1.86	2.17E-14	4.12E-07
	TAF9	Q16594	1.84	2.17E-14	1.37E-06
	PON1	P27169	1.8	4.78E-08	1.43E-05
	TMEM14C	Q9P0S9	1.79	2.16E-11	5.12E-06
	ANXA3	P12429	1.74	3.20E-12	4.16E-06
	CES1	P23141	1.69	2.17E-14	8.40E-07
	LTF	P02788	1.68	2.17E-14	3.07E-07
	SERPING1	P05155	1.68	5.69E-12	4.43E-06
	RNF130	Q86XS8	1.63	0.000796	1.29E-04
	RAB22A	Q9UL26	1.59	0.00021	8.43E-05
	CD63	P08962	1.56	2.17E-14	4.72E-07
	DEFA3	P59666	1.52	8.30E-14	2.86E-06
	C3	P01024	1.48	2.17E-14	8.85E-07
	IGHG2	P01859	1.46	1.33E-09	8.50E-06
	IGHM	P01871	1.45	2.39E-11	5.18E-06
	GCLC	P48506	1.44	8.90E-11	6.06E-06
	HPX	P02790	1.44	1.00E-06	2.44E-05
	VAT1	Q99536	1.41	2.77E-10	6.96E-06
	SLC29A3	Q9BZD2	1.4	0.0247	5.83E-04
	FGB	P02675	1.38	8.30E-14	2.91E-06
	FGG	P02679	1.37	0.000372	1.00E-04
	GPNMB	Q14956	1.37	6.47E-07	2.25E-05
	F2	P00734	1.36	3.97E-06	3.19E-05
	LCN2	P80188	1.36	4.82E-06	3.32E-05
	GP1BA	P07359	1.33	0.013	4.13E-04
	RAF1	P04049	1.31	5.59E-09	1.04E-05
	RBBP5	Q15291	1.28	5.11E-05	5.76E-05
	PF4	P02776	1.27	0.000104	6.94E-05

GCSH	P23434	1.23	2.78E-10	6.96E-06
LYZ	P61626	1.19	3.77E-12	4.23E-06
FGA	P02671	1.18	0.000144	7.59E-05
MMRN1	Q13201	1.17	0.0382	7.58E-04
IGK	Q0KKI6	1.15	9.50E-07	2.41E-05
EPX	P11678	1.12	4.57E-06	3.28E-05
MPO	P05164	1.11	9.41E-08	1.59E-05
A2M	P01023	1.1	1.63E-09	8.74E-06
ACP5	P13686	1.08	5.52E-11	5.72E-06
CD9	P21926	1.08	0.000314	9.50E-05
ITGA2B	P08514	1.03	9.87E-08	1.60E-05
APOC1	P02654	1.02	1.53E-07	1.73E-05
PM20D2	Q8IYS1	1.02	0.00386	2.31E-04

Table S3. Non-tryptic peptides identified in the LC-MS/MS data

See attached.

Table S4. Differentially expressed peptides containing an asparagine cleavage site

Upregulated in BLM-CM

Gene symbol	Protein ID	Peptide sequence	Log_2 FC (BLM-CM/ctrl)	- Log10 (P-value)	previous AA	next AA	start	end	peptide length	last AA	terminus
VIM	P08670	FAVEAANYQDTIGR	1.37	1.40	N	L	351	364	14	R	N
EEF1A1	P68104	HPGQISAGYAPVLDCHTAHIACK	2.05	2.47	N	F	349	371	23	K	N
AZU1	P20160	PGVSTVVLGAYDLR	1.19	1.45	N	R	74	87	14	R	N
TAGLN2	P37802-2	RGPAYGLSR	2.25	2.56	N	E	25	33	9	R	N
SAMHD1	Q9Y3Z3	TPSAEADWSPGLELHPDYK	2.29	2.07	N	T	25	43	19	K	N
HBB	P68871	VDEVGGEALGR	3.82	3.25	N	L	21	31	11	R	N
UBE2V2	Q15819	LPQPPEGQTYNN	1.54	1.38	K	-	134	145	12	N	C
VDAC1	P21796	LTLSALLDGKN	3.05	2.71	K	V	257	267	11	N	C
RBM3	P98179	NQGGYDRYSGGNYRDNYDN	1.14	1.45	R	-	139	157	19	N	C
CALCRL	Q16602	SIHDIENVLLKPENLYN	2.74	2.51	K	-	445	461	17	N	C
GLUL	P15104	TCLLNETGDEPFQYKN	2.47	4.02	R	-	358	373	16	N	C

Upregulated in medium

Gene symbol	Protein ID	peptide sequence	Log_2 FC (BLM-CM/ctrl)	- Log10 (P-value)	previous AA	next AA	start	end	peptide length	last AA	terminus
APOB	P04114	IAGLSLDFSSK	-2.64	1.62	N	L	1759	1769	11	K	N
LCP1	P13796	ILEEIGGGQK	-1.95	2.77	N	V	506	515	10	K	N
KRT1	P04264	KYEDEINK	-2.34	1.79	N	R	269	276	8	K	N
KRT1	P04264	KYEDEINKR	-2.02	1.76	N	T	269	277	9	R	N
APOB	P04114	MGLPDFHIPENLFLK	-2.69	2.64	N	S	1266	1280	15	K	N

IGHG3	P01860-1	TTPPMLDSDGS FFLYSK	-3.09	4.29	N	L	323	339	17	K	N
IGHG1	P01857-1	WYVDGVEVHN AK	-1.43	2.07	N	T	160	171	12	K	N

Table S5. All newly synthesized proteins identified in the secretome of cDC2s using THRONCAT

Gene symbol	Protein ID	log_forward	log_reverse
B7Z747	B7Z747	4.08	-3.34
CTSL	P07711	3.93	-2.67
CTSZ	Q9UBR2	3.39	-3.23
CTSB	P07858	3.12	-2.35
PSAP	P07602	2.91	-2.53
NUCB1	Q02818	2.24	-2.14
CST3	P01034	2.17	-1.90
CTSD	P07339	2.10	-1.72
KRT19	P08727	2.04	-1.34
B2M	P61769	1.94	-2.20
LYZ	P61626	1.93	-1.57
GRN	P28799	1.86	-1.26
DSP variant protein	Q4LE79	1.65	-1.40
IFI30	P13284	1.63	-1.46
RBP4	P02753	1.52	-0.53
TGFBI	Q15582	1.49	-2.46
CTSH	P09668	1.42	-1.14
CTSS	P25774	1.41	-1.12
FABP5	Q01469	1.08	0.87
ACTB	P60709	1.01	-0.55
PRDX1	Q06830	0.98	-0.02
PPIA	P62937	0.88	0.17
HPX	P02790	0.81	0.84
ARG1	P05089	0.78	0.39
ACP5	P13686	0.77	-0.15
ENO1	P06733	0.49	0.21
KRT10	P13645	0.33	-0.76
H3-3B	K7EK07	0.33	-1.06
DKFZp779H1622	Q68DR3	0.24	-0.08
PKM	P14618	0.22	0.98
ALDOA	P04075	0.21	1.09
AZGP1	P25311	0.20	0.27
H2BC5	P58876	0.19	-0.33
KRT1	P04264	0.14	-1.38
DKFZp686I04196	Q6N093	0.02	0.70
TF	P02787	0.00	0.32
AHSG	P02765	-0.08	0.60
IGHG1	P01857	-0.09	0.32
APOA2	P02652	-0.10	0.68
HBA1	Q86YQ4	-0.11	0.23
GC	P02774	-0.17	-0.05
APOE	P02649	-0.20	0.22
ITIH2	P19823	-0.22	0.71
IGL	Q8TCJ5	-0.22	0.40
APOH hCG_1985715	D9IWP9	-0.24	0.26
VIM	P08670	-0.27	0.56
VTN	P04004	-0.28	0.51
HBB	P68871	-0.29	0.86

C4B	P0C0L5	-0.30	0.05
A0A384MDQ7	A0A384MDQ7	-0.32	0.22
IGK	Q0KKI6	-0.32	0.21
A1BG	P04217	-0.33	0.45
HP	P00738	-0.39	0.46
CP	P00450	-0.43	1.04
Bf	A0A7I6GV05	-0.47	0.65
FLJ00385	Q8NF17	-0.48	0.60
LOC102723407	Q9UP60	-0.49	0.46
C3	P01024	-0.49	0.56
APOA1	P02647	-0.54	0.32
ITIH1	P19827	-0.58	0.24
TTR	P02766	-0.59	0.79
hCG_40889	A0A024R962	-0.68	0.83
SERPINC1	P01008	-0.69	0.95
KNG1	P01042	-0.72	0.33
HSPA1A	P0DMV8	-0.79	0.81
AMBP	P02760	-1.00	1.51

Table S6. Antibodies with corresponding dilutions used in the study

Marker	Fluorochrome	Clone	Dilution	RRID identifier
anti-human CD14	APC	M5E2	1 in 40	(BioLegend Cat# 301808, RRID:AB_314190)
anti-human CD14	APC-H7	MϕP9	1 in 30	(BD Biosciences Cat# 560180, RRID:AB_1645464)
anti-human CD1c	BV421	L161	1 in 30	(BioLegend Cat# 331526, RRID:AB_10962909)
anti-human CD20	FITC	L27	1 in 20	(BD Biosciences Cat# 345792, RRID:AB_2868818)
anti-human CD3	PE	HIT3a	1 in 25	(BD Biosciences Cat# 555340, RRID:AB_395746)
anti-human CD3	FITC	HIT3a	1 in 20	(BD Biosciences Cat# 555339, RRID:AB_395745)
anti-human CD56	FITC	NCAM16.2	1 in 20	(BD Biosciences Cat# 345811, RRID:AB_2868832)
anti-human HLA-DR	PerCP	L243	1 in 30	(BioLegend Cat# 307628, RRID:AB_893566)
anti-human CD86	PE-Cy7	FUN-1	1 in 20	(BD Biosciences Cat# 561128, RRID:AB_10563077)
anti-human IDO1	AF488	#700838	1 in 20	(R and D Systems Cat# IC6030G, RRID:AB_10997134)
anti-human CD19	PerCP	4G7	1 in 30	(BD Biosciences Cat# 345778, RRID:AB_2868806)
anti-human CD5	BV711	UCHT2	1 in 25	(BioLegend Cat# 300644, RRID:AB_2832573)
anti-human CD11c	BV785	3.9	1 in 20	(BioLegend Cat# 301644, RRID:AB_2565779)
anti-human CD163	PE	GHI/61	1 in 25	(BD Biosciences Cat# 556018, RRID:AB_396296)
anti-human CD45	PerCP	HI30	1 in 20	(BioLegend Cat# 304026, RRID:AB_893337)
anti-human CD11b	PE-Cy7	Bear1	1 in 25	(Beckman Coulter Cat# A54822)
anti-human β2-microglobulin	PE-Cy7	2M2	1 in 50	(BioLegend Cat# 316317, RRID:AB_2632830)

Table S7. FACS panels used in the study. Related to Figure 3, Figure 4, Figure 6, and Figure S1-S5.

		Laser	Violet (405nm)	Violet (405nm)	Violet (405nm)	Violet (405nm)	Blue (488nm)	Blue (488nm)	Blue (488nm)	Blue (488nm)	Red (633nm)	Red (633nm)
		Band pass filter	448/45	528/45	715/50	755 LP	527/32	586/42	700/54	783/56	660/10	783/56
**Panel**	**Data in**	**Cytometer**										
**cDC2 toxicity**	fig. S1	BD Verse	CD1c BV421				CD20 FITC	L/D PI	HLA-DR PerCP	CD86 PE-Cy7	CD14 APC	
**cDC2 IDO**	fig. 3B	BD Verse	CD1c BV421	L/D e506			*IDO1 AF488*		CD19 PerCP		CD14 APC	
**Purity**	fig. S2B	BD Verse	CD1c BV421				CD20 FITC	CD3 PE				CD14 APC-H7
**LE28/BMV109**	fig. 4C-D, fig 6, S3C-E, S5D-F	BD Lyric	CD1c BV421	L/D e506	CD5 BV711	CD11c BV785	CD3/20/56 FITC	CD163 PE	CD45 PerCP	CD11b PE-Cy7#	LE28/BMV109 Cy5	CD14 APC-H7
**B2M**	fig. S4B	BD Lyric	CD1c BV421	L/D e506	CD5 BV711		CD3/20/56 FITC	CD163 PE	CD45 PerCP	B2M PE-Cy7	CD14 APC	

* Intracellular staining is *italized* and in green #only used for CUSA material (n=1)

Table S8. Patient information

ID	Disease (stage)	Age at inclusion (yrs)	Sex	Used for:	Medication
II-C-12	Melanoma (IIA)	42.9	m	Fig. 4D, 6E, S4	Samples were taken prior to patients receiving surgery and DC vaccinations (treatment was part of a clinical trial)
II-D-03	Melanoma (IIIA)	64.8	m	Fig. 4D, 6E, S4	
II-E-11	Melanoma (IB)	47.1	m	Fig. 4D, 6E, S4	
II-C-14	Melanoma (IIIB)	64.3	f	Fig. 6E, S4	
II-E-02	Melanoma (IIIC)	38.7	m	Fig. 4D, S4	
II-E-03	Melanoma (IIIA)	45.0	m	Fig. 4D, S4	
II-E-04	Melanoma (IIIA)	41.9	m	Fig. 4D, 6E, S4	
CUSA patient	Melanoma (stage IV, M1d) with brain metastasis	70	m	Fig. 6F	anti-PD-1, anti-CTLA-4, dexamethasone

Supplementary figures

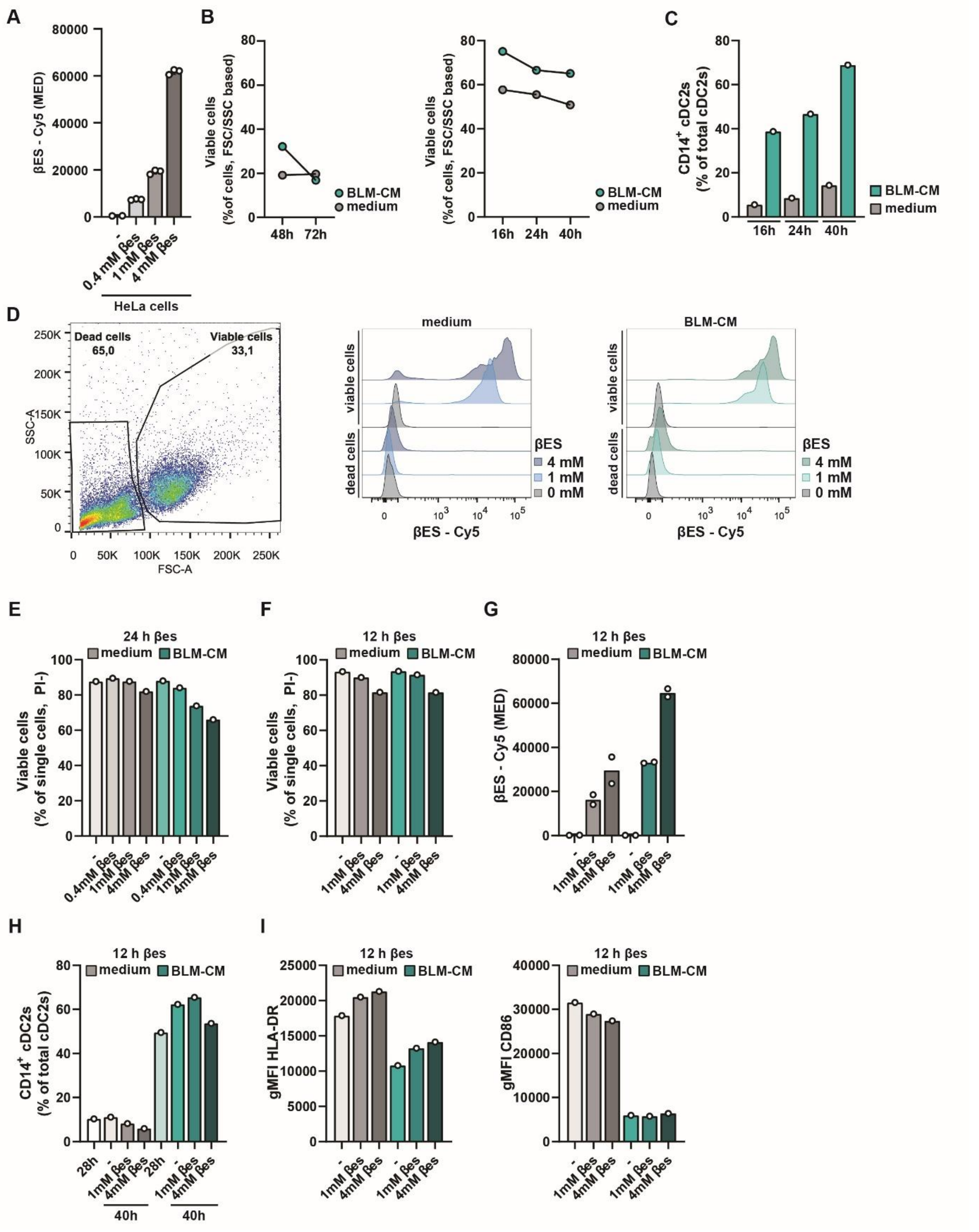

Fig. S1. Optimization of βES labeling in primary human cDC2s during tumor-induced conversion towards DC3s

A) Median (MED) fluorescent signal of βES-Cy5 after 1 h of metabolic labeling with increasing concentrations of βES for HeLa cells in DMEM medium. **B)** Assessment of viability during several culture durations (based on forward scatter (FSC) and side scatter (SSC), which is altered upon cell death, leading to a clearly separated dead cell population as can be seen in D, left). Viability of cDC2s

cultured with 50% BLM-CM or no BLM-CM (medium condition) was higher with shorter culture times while still inducing 47% $CD14^+$ cDC2s within 24 h, and up to 69% $CD14^+$ cDC2s after 40 h **(B,C)**. To assess possible interference of dead cells in βES labeling, we conjugated azide-Sulfo-Cy5 via copper-catalyzed azide-alkyne cycloaddition to intra-cellular βES and measured the fluorescent signal by flow cytometry. As shown in **D)**, dead cells did not show βES- Cy5 signal. To assess toxicity of βES, DCs were labeled for 24 h or 12 h with increasing concentrations, during a total culture period of 40 h **(E-I)**. βES labeling for 24 h largely affected cell viability (14%-22%) **(E)**, whereas 12 h βES labeling reduced the frequency of viable cells with 2% and 12% for 1 mM and 4 mM βES, respectively **(F)**. Increasing the βES concentration from 1 mM to 4 mM enhanced the βES-Cy5 signal **(G)** but was accompanied by increased toxicity **(F)** and effects on phenotype **(H, I)**. Thus, βES toxicity and kinetics of BLM-CM induced conversion to DC3s dictates a total culture period of 40 h, consisting of minimally 24 h with BLM-CM to induce ≥ 50% DC3s prior to labeling for 12 h with 1 mM βES. Therefore, culture with BLM-CM for 28 h followed by labeling with 1 mM βES for 12 h was chosen as final timeline.

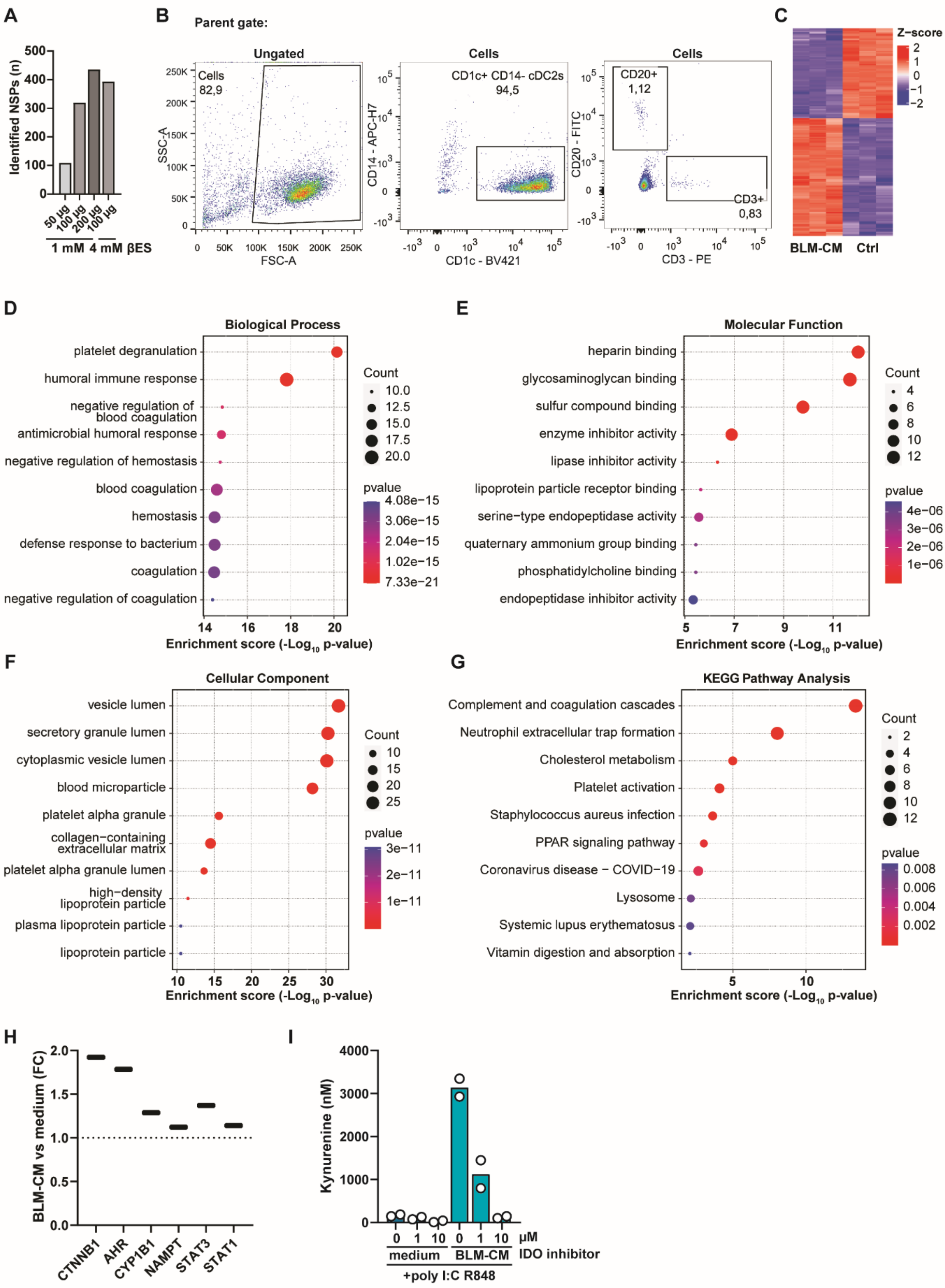

Fig. S2. THRONCAT in primary human monocytes and cDC2s proteomics

A) Monocytes were cultured for 28 h prior to the addition of 1 mM or 4 mM βES and subsequent incubation for 12 h. Cells were washed with PBS, harvested, and counted. Depicted cell amounts in Table S1 were transferred to Eppendorf tubes. Cell pellets were washed twice with PBS, snap frozen in liquid nitrogen and stored at -80 °C until cell lysis. From the whole cell lysates 50 µg, 100 µg, and

200 µg protein (labeled with 1 mM βES) and 100 µg protein (lysate labeled with 4 mM βES) was used as starting material for the βES pull-down enriching newly synthesized proteins (NSPs). NSPs were digested and subjected to LC-MS/MS analysis to identify the number of proteins detectable with the different input amounts. **B)** Representative flow cytometry purity plots after isolation of $CD14^-$ cDC2s from apheresis material. Cells were stained with primary directly labeled antibodies: CD1c-BV421, CD20-FITC, CD3-PE and CD14-APC-H7. **C)** Heatmap visualizing differentially expressed proteins (n = 157) detected in each triplicate for BLM-conditioned medium (CM) and X-VIVO medium control (Ctrl), by LC-MS/MS analysis. **D-G:** Differentially upregulated proteins in medium conditions were subjected to Gene Ontology enrichment analysis for Biological Process **(D)**, Molecular Function **(E)**, Cellular Components **(F)**, and KEGG pathway enrichment analysis **(G)**, all performed by SRplot. Top ten terms are displayed as bubble plots based on p-value. N=1, five individual donors pooled. **H)** Fold change (FC) of several proteins detected in DCs cultured with BLM-CM versus medium control. CTNNB1, AHR, CYP1B1, and STAT3 are implicated in IDO1 signaling, NAMPT and STAT1 are controls that are not (34,35,64). **I)** Kynurenine levels in 48 h supernatants of 50 000 DCs measured by HPLC as measure of IDO activity. DCs were cultured in 100 µL medium or medium with 50% BLM-CM containing 10 mM L-tryptophan, increasing concentrations of the IDO inhibitor Epacadostat, and 20 µg/mL poly I:C and 4 µg/mL R848. Each circle represents an individual donor, bars represent mean values (N=2 independent experiments, ratio paired T-test between kynurenine levels of stimulated DC2 and ti-DC3, without inhibitor, results in P=0.0207). IDO1, indoleamine-2,3-dioxygenase 1; CTNNB1, Catenin beta-1; AHR, Aryl hydrocarbon receptor; CYP1B1, Cytochrome P450 Family 1 Subfamily B Member 1; NAMPT, Nicotinamide phosphoribosyltransferase; STAT, Signal transducer and activator of transcription; LC-MS/MS, liquid chromatography-tandem mass spectrometry. See also figure 2 and 3.

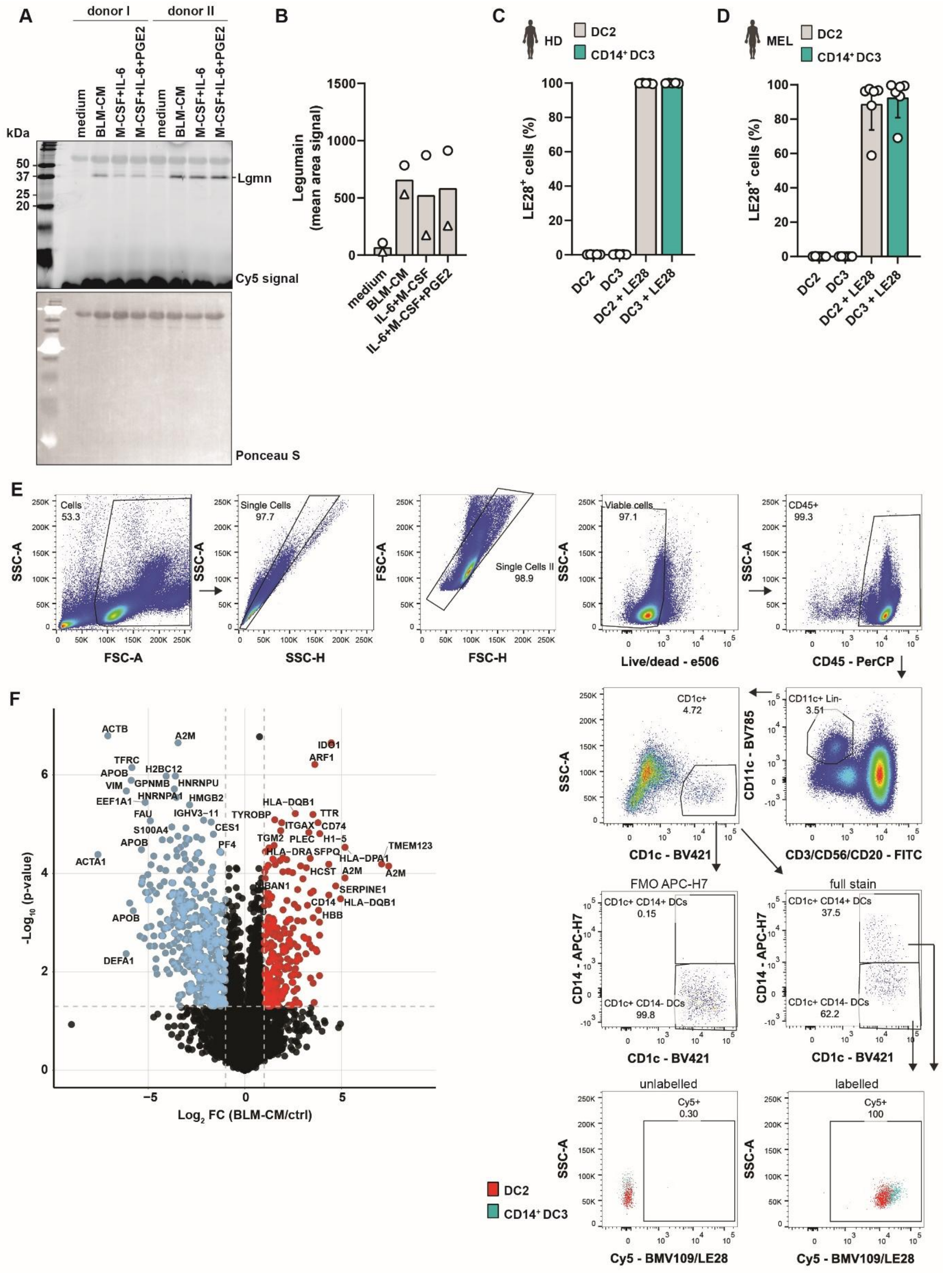

Fig. S3. Legumain activity in primary human dendritic cells

A) Complete SDS-PAGE showing in-gel fluorescent signal of LE28, accompanied by Ponceau S staining of the membrane to which all proteins were transferred. **B)** Protein signals were quantified and corrected for background signal in ImageJ, followed by total protein normalized based on the Ponceau S staining signal. Triangle and circle depict donor I and II, respectively. Bars represent means. **C+D)**

PBMCs from healthy donors (HD) **(C)** or melanoma (MEL) patients **(D)** were labelled with the selective activity-dependent legumain probe LE28 for 1 h at 37 °C or left untreated, followed by analysis by flow cytometry. Shown are the frequencies of labelled cells for N=5 biological replicates for HD and N=6 for MEL patients (mean±SD). **E)** Flow cytometry gating strategy for the analysis of probe LE28 and BMV109 in DC subsets. **F)** Volcano plot displaying the $\log_2$ fold change against $-\log_{10}$ statistical p-value for all non-tryptic peptides detected in the proteomics analysis. Colored dots indicate significant DEPs with p-adjusted < 0.05 and |FC| > 1. See also figure 4.

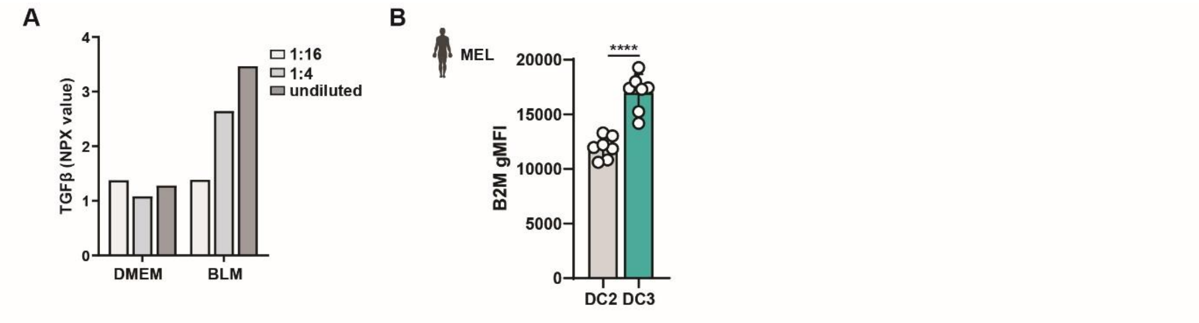

Fig. S4. Follow-up investigation of detected proteins TGFBI and B2M

A) Scaled NPX values of transforming growth factor beta (TGFβ) in several dilutions of 24 h serum-free conditioned medium from BLM cells, measured using the Olink Target 96 Immuno-Oncology panel. **B)** Cell surface expression levels of B2M measured by flow cytometry using directly conjugated anti-human β2-microglobulin-PE-Cy7 (table S5) on PBMCs of melanoma patients. Each symbol shows geometric mean fluorescent intensity (gMFI) of a biological replicate with N=7 (mean±SD, paired T-test).

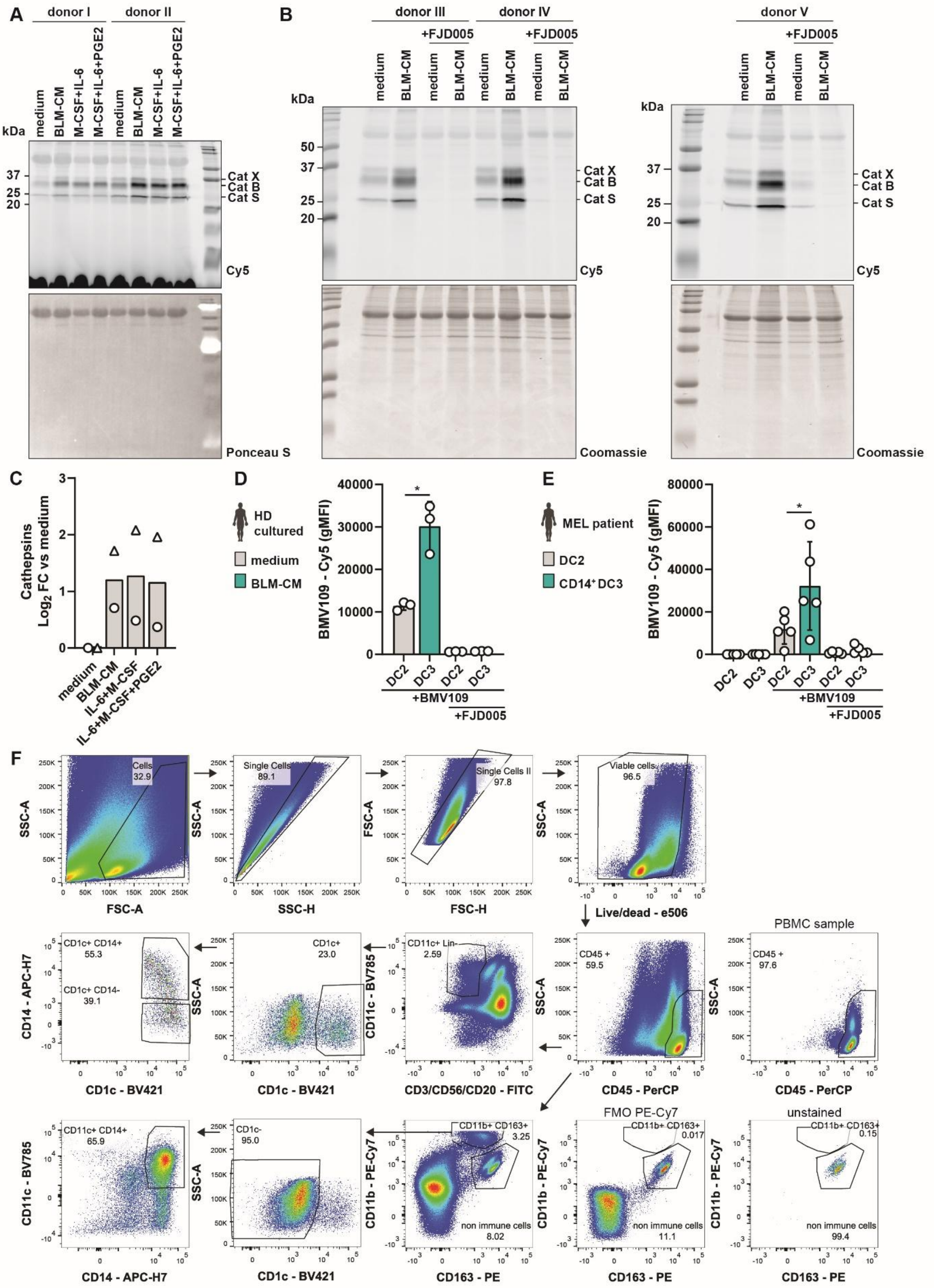

Fig. S5 Cathepsin activity in primary human dendritic cells

A+B) Complete SDS-PAGE showing in-gel fluorescent signal of the activity-based probe BMV109 for all cDC2 donors after culture in the depicted conditions. Gels are accompanied by Ponceau S staining of

the membrane to which all proteins were transferred **(A)** or total protein staining by Coomassie **(B)**. Protein signals were quantified and corrected for background signal in ImageJ, followed by total protein normalized based on the Ponceau S staining signal. Corrected signal intensities were used to calculate log2 normalized fold change (FC) versus medium per individual donor (Figure 6B and **(C). D)** Fractions of the cDC2 samples shown in (B) were acquired by flow cytometry to quantify the BMV109-Cy5 signal (n=3). **E)** PBMCs from melanoma (MEL) patients were labelled with BMV109 in the presence or absence of the cathepsin inhibitor FJD005 for 1 h at 37 °C, or left untreated, followed by analysis by flow cytometry. Each symbol shows geometric mean fluorescent intensity (gMFI) of a biological replicate (n=5, mean±SD). **F)** Gating strategy to analyze DC2 ($Lin^-CD11c^+CD1c^+CD14^-$), DC3 ($Lin^-CD11c^+CD1c^+CD14^+$), and macrophages ($CD11b^+CD163^+CD1c^-CD11c^+CD14^+$), from CUSA tissue samples. Control samples including the PBMCs sample (pure lymphocytes, no tumor cells) and a fluorescence-minus-one (FMO) for PE-Cy7 were used to exclude non-immune cells from $CD11b^+CD163^+$ immune cells. The unstained shows the high autofluorescence of non-immune/tumor cells. See also figure 6.
